# Chemical Diversity and Therapeutic Effects of Essential Oils of *Aniba* Species from the Amazon: A Review

**DOI:** 10.3390/plants10091854

**Published:** 2021-09-07

**Authors:** Rafaela C. S. da Trindade, Júlia Karla A. M. Xavier, William N. Setzer, José Guilherme S. Maia, Joyce Kelly R. da Silva

**Affiliations:** 1 Programa de Pós-Graduação em Biotecnologia, Instituto de Ciências Biológicas, Universidade Federal do Pará, Belém 66075-900, Brazil; rafacabral.bio@gmail.com; 2 Programa de Pós-Graduação em Química, Universidade Federal do Pará, Belém 66075-900, Brazil; julia.xavier@icen.ufpa.br (J.K.A.M.X.); gmaia@ufpa.br (J.G.S.M.); 3 Department of Chemistry, University of Alabama in Huntsville, Huntsville, AL 35899, USA; wsetzer@chemistry.uah.edu; 4 Aromatic Plant Research Center, 230 N 1200 E, Suite 102, Lehi, UT 84043, USA; 5 Programa de Pós-Graduação em Química, Universidade Federal do Maranhão, São Luís 65080-805, Brazil

**Keywords:** *Aniba* spp., Lauraceae, benzenoids and phenylpropanoids, monoterpenes and sesquiterpenes, biological properties

## Abstract

Lauraceae families have great diversity in the world’s tropical regions and are represented mainly by aromatic shrubs and trees with significant production of essential oils (EOs). This work presents a review of the EO chemical profiles from specimens of *Aniba*, including their seasonal variations, geographical distributions, and biological activities in the Amazon biome. Based on the survey, 15 species were reviewed, representing 167 oil samples extracted from leaves, twig barks, and woods. Brazilian Amazon was the most representative geographic area in the number of specimens, highlighting the locations Belém, (Pará state, PA) (3 spp., 37 samples), Santarém (PA) (3 spp., 10 samples), Carajás (PA) (3 spp., 7 samples), and Manaus (Amazonas state, AM) (3 spp., 16 samples). The main compound classes identified in oils were benzenoids and phenylpropanoids, represented by 1-nitro-2-phenylethane, benzyl salicylate, benzyl benzoate and methyleugenol, along with terpenoids, especially monoterpenes and sesquiterpenes, such as linalool, α-phellandrene, β-phellandrene, β-selinene, and spathulenol. The EOs from *Aniba* showed considerable variation in the chemical profiles according to season and collection site. The hierarchical cluster analysis classified the samples into two main groups according to chemical composition. This review highlights its comprehensive and up-to-date information on history, conservation, traditional uses, chemosystematics, pharmacological potential of *Aniba* species.

## 1. Introduction

The genus *Aniba* Alblet (1775) belongs to the Lauraceae family, considered one of the most primitive of the Magnoliids clade [1], and includes 48 accepted species, 25 of which occur in the Brazilian Amazon [2]. The genus originated in the Amazon because the center of species diversity is in the region of the Guianas and Central Amazon, spreading over the humid tropical plains, Antilles, Guyana, and Andes region, without occurrence in Central America [3]. In Brazil, they occur in regions with high rainfall, such as in the Amazon and dry areas in the central and southern regions of the country, with diverse phytophysiognomy such as ombrophilous forests, savannas, canga, and restinga vegetation [2,4].

The first records known about this genus are from an expedition made by Aublet through French Guiana between the years 1762 and 1764, in which the species *Licaria guianensis* Aubl. (1775) was registered in reference to the name “likari”, a tree named by the Galibis Indians. However, Aublet gave this name without having analyzed the fertile parts of the plant. Later, Koeller suggested that it was *Ocotea caudata* (Nees) Mez, which was circumscribed by Mez in 1888, as *Aniba parviflora* (Meisn.) Mez (1889). In 1926, naturalist Adolfo Ducke analyzed the same botanical material collected in the Oiapoque and classified the plant as *Aniba rosaeodora* Ducke. However, when comparing this material with another collected in Juruti Velho (PA, Brazil), Ducke made sure that they were different species, then it was proposed as the *A. rosaeodora* var. *amazonica* Ducke. Later, it was raised to the category of species, as *Aniba duckei*, by Kostermans in 1938 [5]. After reviewing the *Aniba* genus, [3] proposed that *A. duckei* Kosterm. and *A. rosaeodora* var. *amazonica* Ducke were synonymous with *A. rosaeodora* Ducke (1930). 

*Aniba* species are generally large to small trees and rarely shrubs (*A. lancifolia* Kubitzki and Rodrigues). The presence of lenticels is common in the trunk, and when cut, it emits a strong odor, often observed in other parts of the plant, including herborized material due to the presence of oil cells. *Aniba* species have penninerved and alternate leaves, some presented leaves grouped at the top of the branches, and others are distributed equally in the branches. Inflorescences are usually panicles or botryoids containing hermaphrodite flowers, mostly small, pedicellate with erect tepals and bracts caducous. The androecium comprises nine fertile stamens and two valves, with fillets generally the same width as the anthers. The floral tube is conspicuous, the pistil slender, and the ovary ellipsoid or ovoid, glabrous or pilose, included in the floral tube. The ellipsoid or ovoid berry fruit is surrounded by a woody cupule usually containing lenticels [3,6,7,8]. Studies about floral biology showed that most *Aniba* species are strongly protogynous. Some species have asynchronous floral biology events to avoid self-fertilization. The main pollinators are bee species (*Meliponinae*), which recognize pollen receptivity and availability. However, *Aniba* flowers barely open and do not produce nectar, and the pollinators have only pollen as a reward. The fruits possibly have zoochoric dispersion mainly because they serve as food for birds and fish [7]. 

Like the other Lauraceae genera, *Aniba*’s taxonomy needs studies supporting the understanding of the group’s evolution. Species belonging to the genus are considered difficult to identify due to the extreme similarity between them. Thus, morphological [8] and anatomical [9,10,11] studies are essential to identify species and recognize their intraspecific variations. Phytochemical studies have also shown high importance for indicating the chemical variations that occur in the group [12,13], and molecular studies have increasingly helped to understand the genetic diversity of species and the phylogenetic relationships of the group [14,15]. Recently, the combination of phylogenetic data and secondary metabolites of *Aniba* species was evaluated. The chemical composition of essential oils and DNA sequences of *matK*, *psbA*-*trnH*, *rbcL*, and ITS regions of the species *A. rosaeodora*, *A. parviflora*, *A. terminalis* Ducke, and *A. canelilla* (Kunth) Mez showed close relationships according to their chemical and genetic aspects comparable to the traditional morphological classifications [16]. Thus, the combination of data from different areas of knowledge, complemented by information on geographic distribution [17], has revealed coherent information about the group’s evolution [3]. However, the number of existing studies is not proportional to the great diversity of species within the genus, which are indispensable to understanding the evolutionary history, improved classification, and contributing to the conservation and management of *Aniba* species.

The secondary metabolites in *Aniba* extracts are characterized by neoligans, pyrones, benzophenones, allylphenols, and flavonoids [18,19,20]. The homogeneity of the genus is indicated by benzoyl esters and their derivatives and by the benzyltetrahydroisoquinoline alkaloids, practically ubiquitous in all analyzed species [20,21], which permit them to contribute to the chemosystematics of the genus [18]. 

*Aniba* species are excellent producers of essential oils (EOs), and from them, extensive chemical studies have been reported, resulting in the establishment of three groups according to their chemical nature and primary components. Group I, linalool: *A. duckei* and *A. rosaeodora*; group II, benzyl benzoate: *A. burchellii* Kosterm., *A. fragrans* Ducke, *A. firmula* (Nees and Mart. ex Nees) Mez, *A. gardneri* (Meisn.) Mez, *A. guianensis* (Aubl.), *A. parviflora*, and *A. permollis* (Nees) Mez; group III, alkylbenzenes: *A. canelilla*, *A. hostmanniana* (Nees) Mez and *A. pseudocoto* (Rusby) Kosterm. [12,13]. 

The EOs of *Aniba* are rich in volatile compounds that, when isolated or in synergy, presented several biological properties. The EO of *Aniba canelilla* (Kunth) Mez, known as “casca-preciosa,” is rich in 1-nitro-2-phenylethane (50–90%) and methyleugenol (5–40%) and stands out in terms of its cardiovascular and cytotoxic potential [22,23,24]. The EOs of *Aniba duckei* Kosterm and *A. rosaeodora* Ducke, known as “pau-rosa” in the Brazilian Amazon region, display a significant content of linalool, with about 85% [25,26], where both species exhibit remarkable antifungal and cytotoxic activities [27,28,29]. *Aniba parviflora* (Meisn) Mez., popularly called “macacaporanga” or “louro-rosa”, is often confused with *A. duckei* and *A. rosaeodora*, the “pau-rosa” (rosewood) plants. Despite the similarity, these species have distinct aromas in their wood and leaf oils [30,31]. Additionally, *A. parviflora* oil contains a lower percentage of linalool, about 40% [25,26]. Among the several biological activities, the *A. parviflora* oil stands out for presenting a significant antimicrobial potential [32,33,34,35]. 

Considering the *Aniba* species diversity and its predominant occurrence in the Amazon biome, this study aims to present a broad and updated review of research on this plant group’s chemical composition and biological activity.

## 2. Economic and Traditional Uses and Conservation of *Aniba* Species

Several *Aniba* species occurring in the Amazon region present significant economic value and great ecological importance in their native locations [36]. Many of these species are raw materials in public markets of medicinal plants, food, cosmetics, and regional perfumes, and suppliers of good quality wood. Additionally, most of them are essential oil producers, with high value in the national and international markets [27,37].

*Aniba terminalis* Ducke and *A. firmula* have wood with a rigid structure suitable for carpentry and joinery [38]. *Aniba canelilla* is considered hardwood because it is resistant to fungi and xylophagous insects and has good impermeability in naval and civil carpentry. In addition, all parts of *A. canelilla* are aromatic, used as seasonings and ingredients for local dishes, fragrances, and flavoring sachets for clothes [27,38]. *Aniba parviflora* is also known for its quality wood and is used in the perfumery industry, sometimes confused with *A. rosaeodora*, both showing the linalool characteristic aroma, which for a long time have served as ingredients in fragrances and flavor for food and soft drink products [39,40,41].

Traditional Amazonian populations customarily use *Aniba* species to treat diseases and in religious rituals. For example, the leaves and woods of *A. fragrans* and *A. rosaeodora* are used in many Amazonian folk baths, such as the São João festival [42]. *Aniba rosaeodora* essential oil has been used in aromatherapy and home treatments for skincare and the immune and nervous systems [40,43]. In Santarém communities (Pará state), an *A. fragrans* bark decoction is orally used to treat snakebite victims [44]. *Aniba canelilla* powdered seeds are used as an antidiarrheal, and its bark infusion is used to treat coughs as an antispasmodic and stimulant for the central nervous system. Additionally, the *A. canelilla* bark tea is used to treat fever, headache and stomachache by Rorainópolis (Roraima state) and Novo Airão (Amazonas state) communities, located near the Jauaperi River [45,46,47,48]. Additionally, the Indians of Rio Negro (Amazonas state) use the *A. canelilla* bark tea as a stimulant, digestive, antispasmodic tonic and for the treatment of anemia, while the Xipaya, an ethnic Indian group of Altamira (Pará state), utilize the same bark tea as a tranquilizer [49,50].

The aromatic characteristics of some *Aniba* species are mainly due to the presence of linalool, and the *A. rosaeodora* trunkwood is the primary source in the Amazon region, with a linalool content of about 80–97% [51]. However, due to the depletion of trees accessible for commercial exploration, it is usually replaced by other *Aniba* species, which causes variations in their oil yield, between 0.7% and 1.2%. In addition, samples derived from oils of different populations have shown substantial variation in the physicochemical properties and fragrance of the oils, suggesting high genetic variation in the specimens or adulteration resulted from a mixture of other *Aniba* oils [36].

Extractivism is the main activity for the commercial exploitation of aromatic plants from the Amazon. Many species are now under pressure from exploitation, deforestation, and habitat burning [52]. Predatory exploitation and destruction of natural habitats of species with restricted distribution, like some *Aniba* species, has led to the inclusion of several species in the Red List of Threatened Species [53] and the Brazilian Flora Red List [17,54]. From the species surveyed in this review, only *A. canelilla* and *A. rosaeodora* are included in local management programs and subject to ex-situ conservation. Concerning in-situ conservation in protected areas, only *A. canelilla* and *A. parviflora* are listed within the genus [53]. The conservation status of *Aniba* species sampled for the study of chemical composition and biological activity, raised in this review, points out that all of them are in a situation of mostly minor concern, except *A. rosaeodora*, which is endangered due to decades of predatory exploitation that this species has been facing, as the destruction of its natural habitats by logging, livestock, and agriculture, which has culminated in the continued decline of its natural population [54].

Studies have shown that the density of rosewood trees in the forest is low; about 1 tree per 7 hectares [55]. Even so, the rosewood oil intended for trade is obtained exclusively by steam distillation of trunk wood and bark from *A. rosaeodora* trees, consisting of a predatory and a high-risk method of reduction in genetic variability of the species [56]. The indiscriminate cutting of many trees of reproductive age has prevented natural regeneration, leading to a drastic reduction in natural populations, which permitted the Brazilian Institute for the Environment and Natural Resources (IBAMA) to include it in the list of endangered species [57]. Consequently, IBAMA promulgated a set of rules, allowing for the extraction and controlled commercialization of rosewood from the Amazon, only through the preparation and approval of sustainable management and reforestation plans [58]. Rosewood essential oil industry has long been threatened by the scarcity of raw materials and increased environmental regulatory requirements to prevent species extinction [56]. The main limitations for developing production technologies for the species occur because their natural regeneration is irregular and infrequent. Although the propagation by cuttings has a survival rate of about 70%, the availability of matrices for the production of seedlings on a large scale is limited [59,60]. Other limiting factors are the scarcity of information on natural variability, ecology, and distribution of the species [17]. In addition, there is a difficulty for *A. rosaeodora* to produce seedlings. Rosewood propagates naturally through seeds, but these are often preyed upon by birds and insects before maturation [61] and by rodents after maturation [62]. 

A project sponsored by the Benchimol award in 2005 was implemented to guarantee the sustainable supply of rosewood oil in the Brazilian Amazon [56]. As part of the proposal, a germplasm collection of *A. rosaeodora* and other *Aniba* species was created. Based on this, tissue culture studies were carried out, which demonstrated that the rosewood could be propagated satisfactorily in vitro from the cultivation of its stem apices [63]. These activities aimed to facilitate researchers’ access to plant material and reintroduce representative germplasm in regions where the species had already been extirpated, aiming at its in vivo conservation. The researchers of the project highlighted that the articulation of the research sector, government agencies, and the productive sector, represented by distilleries, riverside communities, and small producers, was indispensable for the development of an efficient model of propagation and production of seedlings on a large scale, in order to restore populations in their natural environment [56]. 

## 3. Scope of Collected Data

**Figure 1:**
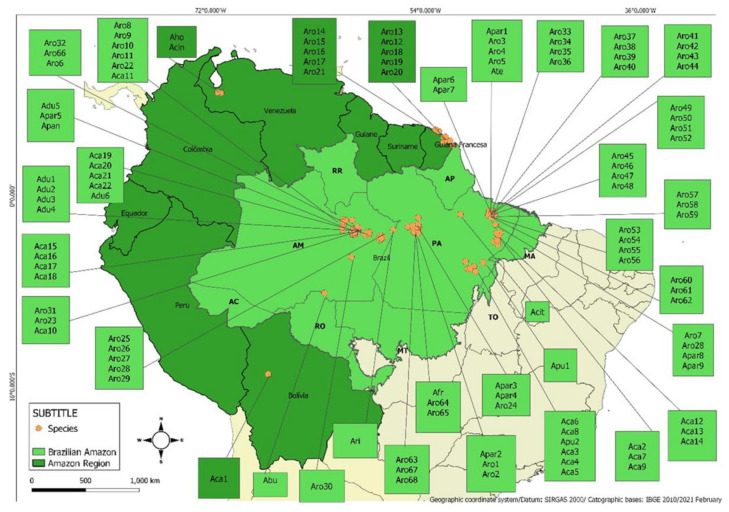
Geographical distribution from specimens of *Aniba* in the Amazon biome, based on its studies of essential oils. The authors built this map using the information of the collection sites available in the bibliographic references for each access. *Aniba burchellii* (Abu), *A. canelilla* (Aca1-Aca22), *A. cinnamomiflora* (Acin), *A. citrifolia* (Acit), *A. duckei* (Adu1-Adu6), *A. fragrans* (Afr), *A. hostmanniana* (Aho), *A. panurensis* (Apan), *A. parviflora* (Apar1-Apar-9), *A. puchury-minor* (Apu1, Apu2), *A. riparia* (Ari), *A. rosaeodora* (Aro1-Aro68), *A. terminalis* (Ate). Abbreviation list: AC: Acre, AM: Amazonas, AP: Amapá, MA: Maranhão, MT: Mato Grossso, PA: Pará, RR: Roraima, RO: Rondônia, TO: Tocantins.

In this review, data collection of *Aniba* species was performed electronically, based on published articles, conference proceedings, theses, and ethnobotanical textbooks. The research was carried out in the Google Scholar, Science Direct, Scopus, and PubMed databases focused on chemical diversity and biological activities of essential oils of *Aniba* species. The keywords used were “essential oils”, “chemical profile”, “biological activity”, “chemical diversity”, “chemical markers of *Aniba* species”. The authors built the map of sample distribution based on the information of the collection sites, available in the bibliographic references to each access (see Figure 1). Based on the survey, there are reports on the species *Aniba burchellii* Kosterm., *A. canelilla* (Kunth) Mez, *A. cinnamomiflora* C.K. Allen, *A. citrifolia* (Nees) Mez, *A. duckei* Kosterm., *A. fragrans* Ducke, *A. gardneri* (Meisn.) Mez, *A. guianensis* Aubl., *A. hostmanniana* (Nees) Mez, *A. panurensis* (Meisn.) Mez., *A. parviflora* (Meisn) Mez., *A. puchury-minor* (Mart.) Mez., *A. riparia* (Nees) Mez., *A. rosaeodora* Ducke, and *A. terminalis* Ducke, corresponding to 167 samples of essential oils.

*Aniba* species showed geographic distribution in four countries of the Amazon biome: Brazil, Bolivia, Venezuela, and French Guiana. The most representative geographic area in specimen number was Brazilian Amazon with highlight to Pará State (67 samples) and Amazonas State (35 samples), predominantly in the cities of Belém (PA) (3 spp., 37 samples) and Manaus (AM) (3 spp., 16 samples), respectively. *Aniba rosaeodora* (68 samples) and *A. canelilla* (22 samples) were the species with the most significant number of studies, followed by *A. parviflora* (9 samples) and *A. duckei* (6 samples). Additionally, studies on EO samples extracted from *A. cinnamomiflora* and *A. hostmanniana* were found only for specimens collected in Venezuela. 

## 4. Multivariate Statistical Analysis Based on the Essential Oils of *Aniba* Species

**Figure 2:**
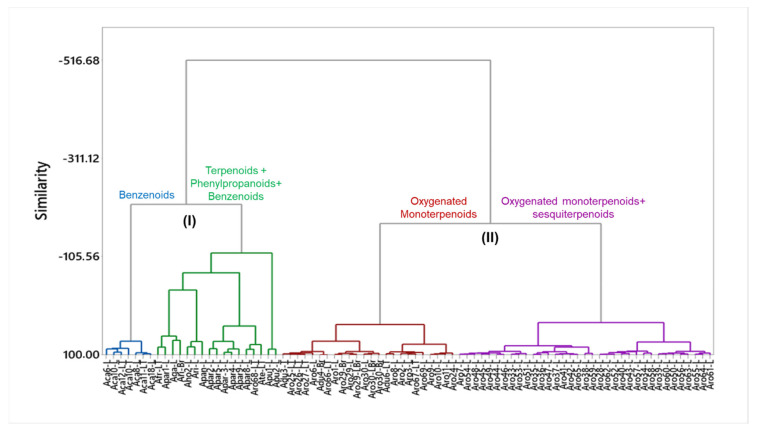
Hierarchical Clusters Analysis (HCA) obtained by Ward Linkage Method to the *Aniba* species based on compound class present in the essential oils extracted from leaves, thin twigs, and branches: *Aniba canelilla* (Aca6-L, Aca8-L, Aca10-L, Aca10-T, Aca11-LT, Aca12-LT, Aca18-L), *A. duckei* (Adu3-T, Adu4-Br, Adu6-LT), *A. fragrans* (Afr-LT), *A. gardneri* (Aga-L), *A. hostmanniana* (Aho2-L), *A. panurensis* (Apan-L), *A. parviflora* (Apar1-L, Apar2-L, Apar-3-L, Apar4-L, Apar5-L, Apar8-L, Apar9-L), *A. puchury-minor* (Apu1-L, Apu2-L), *A. riparia* (Ari-L, Ari-Br), *A. rosaeodora* (Aro1-L, Aro2-L, Aro3-L, Aro6-L, Aro7-L, Aro8-L, Aro9-L, Aro10-L, Aro11-L, Aro24-L, Aro25-LT, Aro26-LT, Aro27-LT, Aro28-L, Aro29-L, Aro29-Br, Aro29-LBr, Aro30-L, Aro30-Br, Aro30-LBr, Aro33-L, Aro34-L, Aro35-L, Aro36-L, Aro37-L, Aro38-L, Aro39-L, Aro40-L, Aro41-L, Aro42-L, Aro43-L, Aro44-L, Aro45-L, Aro46-L, Aro47-L, Aro48-L, Aro49-L, Aro50-L, Aro51-L, Aro52-L, Aro53-L, Aro54-L, Aro55-L, Aro56-L, Aro57-L, Aro58-L, Aro59-L, Aro60-L, Aro61-L, Aro62-L, Aro63-L, Aro64-L, Aro65-L, Aro66-LT, Aro67-LT, Aro68-LT, Aro69-L, Ate-LT). Abbreviation list: L: leaves, T: thin twigs, Br: branches.

A multivariate statistical analysis was performed to group the compound classes as chemical markers of the *Aniba* species. The EOs from specimens of *Aniba* were divided into two groups according to the tissue: leaf, thin twig, and branch; stem, bark, and trunk wood. Seventy-six specimens of *A. canelilla*, *A. duckei*, *A. fragrans*, *A. gardneri*, *A. hostmanniana*, *A. panurensis*, *A. parviflora*, *A. puchury-minor*, *A. riparia*, and *A. rosaeodora* showed 84 EO samples of leaves, thin twigs, and branches. In contrast, thirty-eight EO samples of stems, barks, and trunk woods of *A. canelilla*, *A. cinnamomiflora*, *A. citrifolia*, *A. gardneri*, *A. guianensis*, *A. parviflora*, *A. puchury-minor*, *A. rosaeodora*, and *A. riparia* were represented by thirty-one specimens (see Figure 2).

Total percentage of the following compound classes, monoterpene hydrocarbons (MH), oxygenated monoterpenes (OM), sesquiterpene hydrocarbons (SH), oxygenated sesquiterpenes (OS), phenylpropanoids (PP), and benzenoids (BZ), present in the leaves, thin twigs, branches, stems, barks, and trunk woods was applied as variables. The data matrix was standardized by subtracting the mean from each compound’s value and then subtracting it by the standard deviation. The values were submitted to Hierarchical Cluster Analysis (HCA) based on Ward binding and Euclidean distance, using the software Minitab 17 (free 390 version, Minitab Inc., State College, PA, USA).

### 4.1. Essential Oils from Leaves, Thin Twigs and Branches of Aniba Species

Based on the dendrogram obtained by HCA, using the classes of compounds as variables, 84 EO from the leaves, thin twigs, and branches of *Aniba* species were classified into two main clusters, presenting a similarity of −516.68%. Cluster I was composed of twenty-four oils of *A. canelilla*, *A. puchury-minor*, *A. gardneri*, *A. hostmanniana*, *A. riparia*, *A. fragans*, *A. parviflora*, *A. rosaeodora*, and *A. terminalis*. The samples of cluster I were divided into two subgroups with a similarity of −214.58%. Subgroup I-1 was formed by seven oils from *A. canelilla* with a high concentration of benzenoids, especially 1-nitro-2-phenylethane (68.7–95.3%), and with a similarity of 71.50%. On the other hand, subgroup I-2 comprised oils rich in terpenoids (traces—89.3%), benzenoids (traces—45.4%), and phenylpropanoids (traces—44.5%) with a similarity of −112.69%. In this I-2 subgroup, seventeen samples of *A. fragrans*, *A. gardneri*, *A. hostmanniana*, *A. parviflora*, *A. riparia*, *A. parviflora*, *A. rosaeodora*, *A. terminalis*, and *A. puchury-minor* were grouped.

In cluster II, sixty samples of *A. duckei* and *A. rosaeodora* were grouped and divided into two subgroups with a similarity of −174.49%. The subgroup II-1 was composed of twenty-four oils of *A. duckei* and *A. rosaeodora* with a similarity of 36.69% and characterized by the high concentration oxygenated monoterpenes, such as linalool (79.0–88.60%). The subgroup II-2 comprised thirty-six oils of *A. rosaedora* rich in oxygenated monoterpenes (57.2%), sesquiterpene hydrocarbons (12.69%), and oxygenated sesquiterpenes (8.74%), showing a similarity of 32.51%. According to the individual species, the disposition of the classes of compounds can be visualized in Figure 2. The information on the main compounds of EOs extracted from leaves, thin twigs, and branches of *Aniba* species, their corresponding collection data, and their extraction method are present in Table 1.

#### 4.1.1. Cluster I: Benzenoid-Rich Oils 

EO samples of *Aniba canelilla* (Aca) collected in Serra dos Carajás (PA, Brazil) (Aca6-L and Aca8-L), Adolpho Ducke Forest Reserve (AM, Brazil) (Aca10-L and Aca10-LT), Ulianópolis (PA, Brazil) (Aca12-LT), and Novo Airão (AM, Brazil) (Aca11-LT) were arranged in the subgroup I-1 (Figure 2). These samples showed a higher similarity level (71.50%) due to a higher concentration of benzenoids, characterized by the significant compound 1-nitro-2-phenylethane (68.2–95.3%). However, small quantities of linalool (5.2–8.8%), eugenol (5.2%), benzaldehyde (4.8%), spathulenol (4.8%), β-selinene (4.5%), and β-caryophyllene (3.5%) also were identified (Table 1) [27,35,64,65].

#### 4.1.2. Cluster I: Terpenoid, Phenylpropanoid and Benzenoid-Rich Oils

Seventeen samples formed subgroup I-2 with significant chemical diversity by their main compounds and a similarity level of −112.69% (Figure 2). The EO of two specimens of *A. puchury-minor*, collected in Serra dos Carajás (PA, Brazil) (Apu1-L and Apu2-L), displayed sesquiterpene hydrocarbons (48.29%) and phenylpropanoids (41.50%) with significant contents. The major compounds were elemicin (23.46% and 21.5%), bicyclogermacrene (15.4%) and germacrene (13.42%) (Table 1) [66,67].

The EO samples of *A. gardneri*, *A. hostmanniana,* and *A. riparia* were rich in sesquiterpene hydrocarbons (4.8–65.5%), oxygenated sesquiterpenoids (10.7–43.5%), and benzenoids (3.2–45.4%) [12,68,69] (Figure 2). The oils of *A. hostmanniana* (Aho2-L) and *A. gardneri* (Aga-L) showed benzyl benzoate (29.3% and 44.1%) and δ-cadinene (12.0% and 4.8%) as the most abundant compounds [12,68]. On the other hand, the majority compounds of *A. riparia* (Ari-Br and Ari-L) were (*E*)-nerolidol (19.4%), β-caryophyllene (16.9%), elemol (16.2%), and α-humulene (14.9%, 10.9%) [69] (Table 1). These specimens were collected in Parintins (AM, Brazil) (Ari-L, Ari-Br), Mérida (Venezuela) (Aho2-L). The *A. gardneri* (Aga-L) was sampled in the Brazilian Amazon but without a collection site mentioned. The oil of *A. panurensis* (Apan-L), collected in Adolpho Ducke Forest Reserve (Manaus, AM, Brazil) was characterized by a high content of sesquiterpene hydrocarbons (89.3%) and β-caryophyllene (33.5%), germacrene-D (25.4%), and α-copaene (7.5%) were most representative constituents [70].

The oils of *A. fragans* (Afr), *A. parviflora* (Apa), *A. rosaeodora* (Aro), and *A. terminalis* (Ate) showed monoterpene hydrocarbons (31.54%), oxygenated monoterpenoids (32.07%), sesquiterpene hydrocarbons (13.75%), and oxygenated sesquiterpenoids (17.72%) as the main compound classes (Figure 2). The most representative constituents were linalool (11.90–45.0%), α-phellandrene (4.1–32.8%), and β-phellandrene (7.55–23.60%) (Table 1). In the EO of *A. fragrans* were linalool (32.4%), spathulenol (19.1%), and limonene (14.5%). The species were collected in the Curuá-Una (PA, Brazil) (Afr-L), Santarém (PA, Brazil) (Apa2-L, Apa3-L, and Apa4-L), Adolpho Ducke Forest Reserve (AM, Brazil) (Apar5-L), Tomé-Açu (PA, Brazil) (Apar8-L and Apar9-L), Arapiuns (PA, Brazil) (Aro68-LT) and Belém (PA, Brazil) (Ate-LT and Apar1-L) [32,33,34,35,51,71,72,73,74,75].

#### 4.1.3. Cluster II: Oxygenated Monoterpene-Rich Oils 

Twenty-four oils of *Aniba duckei* and *A. rosaeodora* were arranged in subgroup II-1, comprising samples collected in Pará and Amazonas state, Brazil, with a similarity level of 36.69% (Figure 2). The *A. rosaeodora* EOs from Pará state showed oxygenated monoterpenes contents varying from 81.12–91.80%. The major compound was linalool (79.0–88.60%), followed of β-selinene (2.0%), aromadendrene oxide (2.5%), (*E*)-nerolidyl acetate (1.5%) and *cis*-linalool oxide (1.84%) (Table 1). These samples were collected in Belém (Aro3-L), Curuá-Una (Aro1-L, Aro24-L), Santarém (Aro2-L), and Rurópolis (Aro67-LT) (PA, Brazil) [33,51,71,74,76]. 

Specimens of *A. duckei* and *A. rosaoedora* collected in the Amazonas state exhibited significant variation in their oxygenated monoterpenes (71.8–98.5%) contents and chemical diversity of the oils. Linalool varied from 71.76% to 93.60%, followed by β-selinene (0.64–6.41%), α-terpineol (1.11–5.6%), spathulenol (0.34–4.0%), caryophyllene oxide (2.0–3.2%), and *cis*-linalool oxide (1.6–3.03%), in smaller proportions (Table 1). The Amazonas collection sites were Itacoatiara (Adu6-LT), Presidente Figueiredo (Aro6-L), Novo Airão (Aro8-L, Aro9-L, Aro10-L and Aro11-L), Maués (Aro25-LT, Aro26-LT, Aro27-LT, Aro29-L, Aro29-Br and Aro29-LBr), Novo Aripuanã (Aro30-L, Aro30-Br and Aro30-LBr), Adolpho Ducke Forest Reserve in Manaus (Adu3-L, AduBr-4 and Aro66-LT) and Aro69-L (collection site not indicated) [25,29,30,77,78,79,80,81,82].

#### 4.1.4. Cluster II: Oils Rich in Oxygenated Mono- and Sesquiterpenes

Subgroup II-2 was represented by thirty-six samples of *A. rosaeodora* oils collected in Tomé-Açu (PA, Brazil) (Aro7-L and Aro28-L), Novo Aripuanã (AM, Brazil) (Aro30-LBr), Belém (PA, Brazil) (Aro33-L to Aro62-L), and Curuá Una (PA, Brazil) (Aro63-L to Aro65-L) (Figure 2). These oils showed a similarity level of 32.51%, and the oxygenated monoterpenes, sesquiterpene hydrocarbons, and oxygenated sesquiterpenes contents were 57.54%, 12.62% and 8.74%, respectively. The major constituents were linalool (38.48–71.05%), spathulenol (3.73–7.20%), and β-selinene (3.79–6.41%) (Table 1) [73,80]. 

### 4.2. Essential Oils from Stem, Bark and Trunkwood of Aniba Species

**Figure 3:**
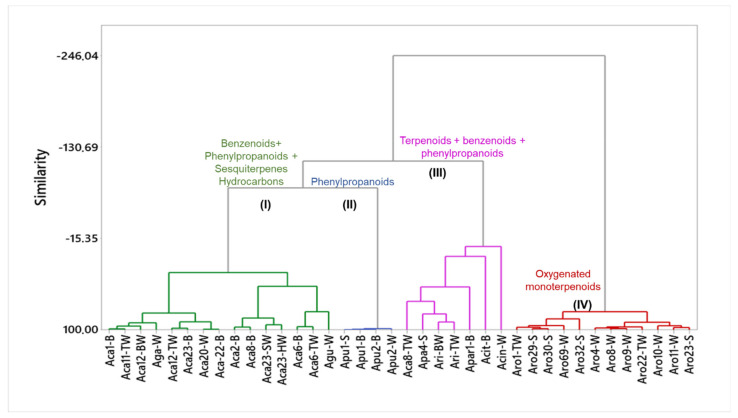
Hierarchical Clusters Analysis (HCA) obtained by Ward Linkage Method to *Aniba* species based on compound class present in the essential oil extracted from barks and woods: *Aniba canelilla* (Aca1-B, Aca2-B, Aca6-B, Aca6-TW, Aca8-B, Aca8-TW, Aca11-TW, Aca12-BW, Aca12-TW, Aca20-W, Aca22-B, Aca23-SW, Aca23-HW, Aca23-B), *Aniba cinnamomiflora* (Acin-W), *Aniba citrifolia* (Acit-B), *A. gardneri* (Aga-W), *A. guianensis* (Agu-W), *A. parviflora* (Apar1-B, Apar4-S), *A. puchury-minor* (Apu1-S, Apu1-B, Apu2-B, Apu2-W), *A. rosaeodora* (Aro1-TW, Aro4-W, Aro8-W, Aro9-W, Aro10-W, Aro11-W, Aro22-TW, Aro23-S, Aro29-S, Aro30-S, Aro32-S, Aro69-W), *Aniba riparia* (Ari-BW, Ari-TW). Abbreviation list: B: bark, S: steam, W: wood, BW: barkwood, HW: heartwood, SW: sapwood, TW: trunk wood.

Based on the dendrogram resulting from the HCA, thirty-eight oils from the stem, bark, and trunkwood of *Aniba* species were classified into four main clusters. Cluster I comprised fifteen oils of *A. canelilla*, *A. gardineri*, and *A. guianensis* rich in benzenoids (34.4–92.5%), phenylpropanoids (traces—65.3%), and sesquiterpene hydrocarbons (traces—15.6%), showing a similarity of 28.2%. Four samples of *A. puchury-minor* were grouped in cluster II, characterized by a high content of phenylpropanoids (99.14%) and a similarity level of 98.65%. Cluster III was composed of seven samples of *A. canelilla*, *A. cinnamomiflora*, *A. citrifolia*, *A. parviflora*, and *A. riparia* with a similarity of −5%. The main classes were benzenoids (traces—47.4%), oxygenated monoterpenes (4.2–45.4%), monoterpene hydrocarbons (traces—43.7%), sesquiterpene hydrocarbons (4.0–33.3%), phenylpropanoids (traces—16.7%), and oxygenated monoterpenes (traces—13.6%), with significant contents. Finally, cluster IV grouped all oil samples of *A. rosaeodora*, presenting a high level of similarity (77.39%). These samples were characterized by significant amounts of oxygenated monoterpenes, especially linalool (81.6–95.0%). According to the related species, the disposition of the classes of compounds can be visualized in Figure 3, and the information on the main compounds of the stem, bark, and trunk wood EOs from *Aniba* species and their collection data, are present in Table 1.

#### 4.2.1. Cluster I: Oils Rich in Benzenoids, Phenylpropanoids and Sesquiterpene Hydrocarbons 

Fifteen oils extracted from *A. canelilla* (Aca), *A. gardneri* (Aga) and *A. guianensis* (Agu) were grouped in cluster I, presenting a similarity of 28.02% (Figure 3). The EOs of *A. canelilla*, collected in Serra dos Carajás (PA, Brazil) (Aca6-B, Aca6-TW and Aca8-W), and *A. guianensis* (Agu-W, collection site not informed) showed benzenoids (34.4–65.4%), phenylpropanoids (8.5–65.3%) and sesquiterpene hydrocarbons (traces—15.6%) as predominant compound classes. 1-Nitro-2-phenylethane (50.2–60.5%), methyleugenol (21.3–34.7%), and β-sesquiphellandrene (5.4–6.4%) were the main compounds presented in *A. canelilla* oil samples. On the other hand, the most abundant compounds of *A. guianensis* oil were benzyl benzoate (59.0%), benzyl salicylate (6.4%), and methyl isoeugenol (5.0%) (Table 1) [12,64]. 

*Aniba canelilla* and *A. gardneri* collected in Fatima de Chimanes (Bolivia) (Aca1-SB), Novo Airão (PA, Brazil) (Aca11-TW), Ulianópolis (PA, Brazil) (Aca12-BW and Aca12-TW), Itacoatiara (AM, Brazil) (Aca20-W, Aca22-B, Aca23-B and Aga-W with not reported collection site) were rich in benzenoids (34.4–92.5%) and phenylpropanoids (traces—65.3%) (Figure 2). In general, the main compounds identified in *A. canelilla* oils were 1-nitro-phenylethane (47.4–92.1%) and methyleugenol (2.0–38.6%). However, high contents of methyleugenol were reported in the sapwood (65.3%) and heartwood (52.9%) tissues of *A. canelilla*. Additionally, significant contents of benzenoids, such as benzyl benzoate (78.1%) and phenylethyl benzoate (14.3%), were identified in the EO of *A. gardineri* (Table 1) [12,27,83,84,85,86,87].

#### 4.2.2. Cluster II: Phenylpropanoid-Rich Oils 

Cluster II was formed by oils of *A. puchury-minor* collected in the Serra dos Carajás (PA, Brazil) (Apu1-S and Apu1-B), and Canaã do Carajás (PA, Brazil) (Apu-B and Apu2-W) (Figure 3). The oils samples showed a similarity of 98.65% due to the high contents of phenylpropanoids (99.14%). The main compounds of the oils were (*E*)-asarone (29.95–52.75%), methyleugenol (17.62–43.10%) and (*E*)-isoelemicin (11.87–23.1%) (Table 1) [66,67].

#### 4.2.3. Cluster III: Oils Rich in Terpenoids, Benzenoids and Phenylpropanoids

The oils of *A. canelilla* (Aca), *A. cinnamomiflora* (Acin), *A. citrifolia* (Acit), *A. parviflora* (Apa) and *A. riparia* (Ari) showed significant chemical diversity for their main compounds, grouped in Cluster III, showing a similarity level of -5.0% (Figure 3). The most representative compound classes were benzenoids (traces—47.4%), oxygenated sesquiterpenes (4.2–45.4%), monoterpene hydrocarbons (traces—43.7%), sesquiterpene hydrocarbons (4.0–33.3%), oxygenated monoterpenes (traces—13.6%) and phenylpropanoids (traces—16.7%). The oil of the trunk wood of *A. canelilla* was rich in benzenoids (47.4%), oxygenated sesquiterpenes (20.5%), and phenylpropanoids (12.5%), showing 1-nitro-2-phenylethane (47.4%), *epi*-α-cadinol (19.9%), and methyleugenol (10.5%) as the main constituents (Table 1) [64]. The oil of *A. cinnamomiflora* from Los Andes (Merida, Venezuela) (Acin-W) showed a high content of the lipid γ-palmitolactone (54.0%), followed by 1-*epi*-cubenol (9.56%) and δ-cadinene (6.05%) [88].

Oils of *A. riparia* (Ari-BW and Ari-TW), collected in Parintins (AM, Brazil), were characterized by the presence of sesquiterpene hydrocarbons (21.7%, 27.2%), benzenoids (39.2%, 44.0%), and oxygenated sesquiterpenes (14.2%, 16.1%). The most abundant compounds were benzyl benzoate (30.9%, 36.2%), terpinen-4-ol (9.3%), benzyl salicylate (7.9%, 7.7%), and *cis*-calamenene (7.2%) (Table 1) [69].

The EO samples of *A. citrifolia* collected from Melgaço (PA, Brazil) (Acit-W) showed safrole (16.7%), α-pinene (10.6%), and β-pinene (11.2%) as the main constituents. Monoterpene hydrocarbons (43.7%), sesquiterpene hydrocarbons (17.8%), and phenylpropanoids (16.7%) were the main compound classes [89]. *Aniba parviflora* oils from Belém (PA, Brazil) (Apar1-B) and Santarém (PA, Brazil) (Apar4-S) showed an abundance of oxygenated sesquiterpenes (33.3% and 24.25%), sesquiterpene hydrocarbons (45.4% and 18.67%), and oxygenated monoterpenes (13.6% and 13.35%). Linalool (11.90%), aristolene (11.07%) and β-eudesmol (3.97%) predominated in the *A. parviflora* oil from Belém (PA), while *γ*-eudesmol (16.8%), β-caryophyllene (15.7%), and linalool (12.4%) were the majority compounds of *A. parviflora* from Santarém (PA) (Table 1) [32,34].

#### 4.2.4. Cluster IV: Oxygenated Monoterpenoid-Rich Oils 

Cluster IV was composed of twelve samples of *A. rosaeodora* from Pará and Amazonas states, with a similarity level of 77.39% (Figure 3). Pará state samples, collected in Curuá-Una (PA, Brazil) (Aro1-TW) and Belém (PA, Brazil) (Aro4-W), exhibited a significant content of oxygenated monoterpenes (91.86%). The primary compound was linalool (87.93%), followed by minor amounts of α-terpineol (2.9%), *cis*-linalool oxide (1.0–1.68%), and *trans*-linalool oxide (0.90-1.60%) [25,76,90,91]. On the other hand, the oils from Amazonas state, collected in Novo Airão (AM, Brazil) (Aro8-W, Aro9-W, Aro10-W, Aro11-W, and Aro22-TW), Maués (AM, Brazil) (Aro29-W), Manaus (AM, Brazil) (Aro23-S and Aro32-S), Novo Aripuanã (AM, Brazil) (Aro30-S) and Aro69-W (collection site not reported), showed a percentage of oxygenated monoterpenes varying from 81.6% to 95.0%. The linalool content ranged from 63.16% to 86.12%, followed by *trans*-linalool oxide (0.90–9.73%), α-terpineol (3.8–5.6%), benzyl benzoate (2.7%), and *cis*-linalool oxide (1.6–2.7%) [25,30,70,80,92,93]. 

## 5. *Aniba* Commercial Samples

Leaf essential oils from *A. canelilla* purchased at Ver-o-peso market (Belém, Brazil) were dominated by 1-nitro-2-phenylethane (99.1%). On the other hand, the trunk wood oil of *A. canelilla*, obtained by a donation from a Manaus oil producer (AM, Brazil), presented 1-nitro-2-phenylethane (68.8%) and a significant amount of methyleugenol (28.1%) [77].

The oils of *A. rosaeodora* obtained from Dr. Josif Pancic, Institute for Medicinal Plants Research (Belgrade, Serbia), Erbamea (Istrana, Treviso, Italy), and Oshadhi Ltd. (Cambridge, UK) showed a total of oxygenated monoterpenes ranging from 51.7% to 92.4%. Linalool (44.1–81.3%) and linalyl acetate (31.4%) were the main compounds, followed by minor concentrations of limonene (19.2%), β-caryophyllene (10.5%), geraniol (7.8%) and α-terpineol (4.78%) [94,95,96]. In other commercial samples, with origins not reported, the oxygenated monoterpenes contents varied from 74.6% to 100%. The most representative compound was linalool (72.0–86.5%), followed by *cis*-linalool oxide (1.06–5.8%), *trans*-linalool oxide (1.1–5.2%), and α-terpineol (4.5%) [76,77,97,98]. 

## 6. Seasonal Variation in the *Aniba* Volatile Constituents

The essential oil chemical composition of *Aniba* species can be influenced by environmental factors, such as light, humidity, soil, harvest time, as well as by oil variation in the plant organs and their stage of development [84,99,100,101]. Different responses in EO production by *Aniba* species can be evaluated to improve the oil productivity in natural or cultivation conditions [102]. 

Seasonal changes influenced the oil yield and chemical composition from different tissues of *A. canelilla* (Aca3, Aca4 and Aca5) collected in the Serra dos Carajás (PA, Brazil) during the rainy and dry seasons. In the leaf EO, 1-nitro-2-phenylethane production was higher in the rainy season (70.6–95.3%) in comparison to the dry season (39.0–42.1%) (Figure 3). Conversely, the bark and trunk wood oils exhibited high contents of 1-nitro-2-phenylethane (69.2–94.3%) followed by low amounts of methyleugenol (1.0–17.7%) in the rainy season, while in the dry season, the content of 1-nitro-2-phenylethane and methyleugenol ranged between 48.6–73.3% and 22.2–45.8%, respectively (Figure 3) [84]. 

**Figure 4:**
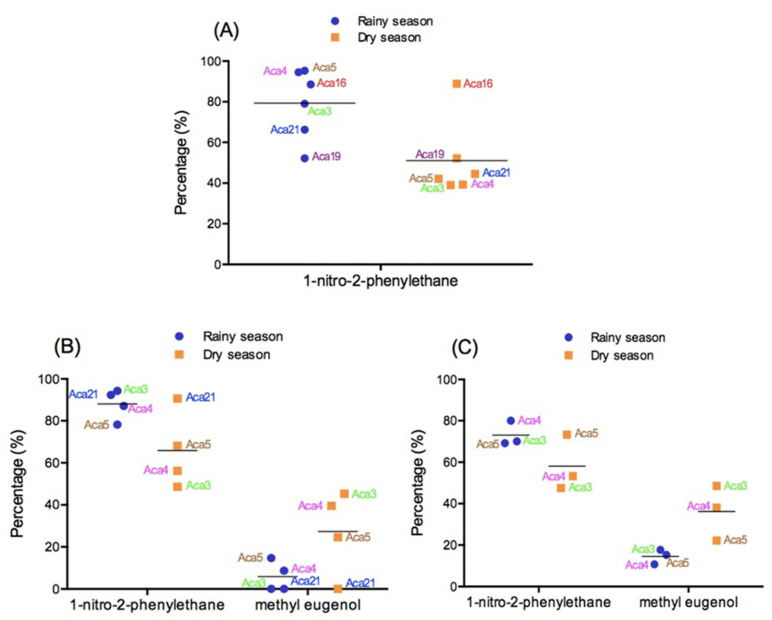
Content variation of 1-nitro-2-phenylethane and methyleugenol in the oils of leaves (**A**) stems and thin twigs (**B**) and trunk wood (**C**) from *Aniba canelilla* (Aca) during seasonal studies.

*Aniba canelilla* leaf EO (Aca16), collected in Manaus (AM, Brazil) during the dry and rainy season, showed similar chemical profiles with contents of 1-nitro-2-phenylethane of 88.5% and 88.9%, respectively (Figure 4) [22]. However, the EO composition from a specimen of *A. canelilla* (Aca19) collected in Itacoatiara (AM, Brazil) changed drastically according to season. The average percentages of 1-nitro-2-phenylethane identified in the leaves and thin twigs of *A. canelilla* were 52.2% and 92.7% in the rainy and dry season, respectively (Figure 4) [102]. In another study, the content of 1-nitro-2-phenylethane in the leaves of *A. canelilla* (Aca21) collected in Itacotiara (AM, Brazil) showed a high variation during the months of collection. The dry season showed variable contents (13.17–74.55%) compared to the rainy season (31.22–84.33%). On the other hand, these abrupt changes of 1-nitro-2-phenylethane were not observed in the stems (Aca21) (Figure 4) [101].

**Figure 5:**
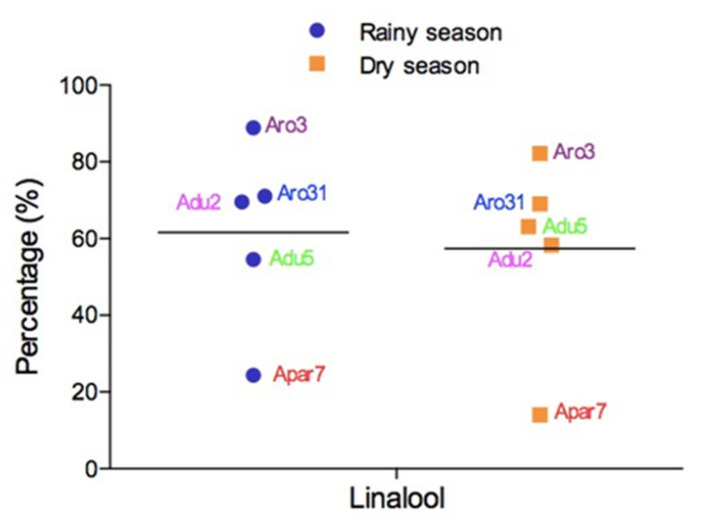
Linalool variation in the leaf oils from *A. parviflora* (Apa), *A. duckei* (Adu), and *A. rosaeodora* (Aro) during seasonal studies.

Linalool production in the leaf oils of two specimens of *A. duckei* (Adu2), collected in Manaus (AM, Brazil), showed significant variations according to season. The leaf oil content was higher in the dry season (62.4–76.69%) than in the rainy season (56.26–60.38%) (Figure 5) [103]. In another study, conversely, a higher percentage of linalool was observed in the rainy season (63.16%) in comparison to the dry season (54.5%) for the leaves of *A. duckei* (Adu5). However, the linalool content of the thin twigs was maintained between 69.38% and 71.98% in the rainy and dry seasons, respectively (Figure 5) [99]. 

Leaf oils of *A. rosaeodora* (Aro3) from Belém (PA, Brazil) exhibited the highest linalool content in the dry season with percentages varying from 86.6% to 96.1%. In the rainy season, the production of linalool varied from 74.8% to 84.3%, except in March, when the percentage decreased to 68.0%, coinciding with more extensive water precipitation (Figure 5) [25]. Essential oils of *A. rosaeodora* (Aro31) from Manaus (AM, Brazil) showed linalool contents of 69.0% (leaves) and 71.0% (branches) in the dry season, compared to 78.0% (leaves) and 84.0% (branches) in the rainy period (Figure 5) [28]. The leaves of *A. parviflora* (Apa7), collected in Belém (PA, Brazil), showed linalool content of 14.07% and 24.37% in the dry and rainy seasons, respectively (Figure 5) [104]. The variation of sesquiterpene content could be a consequence of dilution by increased expression of another component [105]. The seasonal variation of linalool content in the leaves of *A. duckei*, *A. parviflora*, and *A. rosaeodora* can be visualized in Figure 5.

**Table 1:** Main constituents of *Aniba* essential oils from the Amazon.

Species	Collection Site	Plant Part	Extraction Type	Major Components	References
*A. burchellii*	Humaitá, AM, Brazil	Leaf	SD	Benzyl salicylate (45.6%), α-pinene (12,1%), and benzyl benzoate (5.2%)	[106]
*A. canelilla*	Manaus, AM, Brazil	Sapwood	HD	Methyleugenol (65.3%) and eugenol (34.4%)	[12]
*A. canelilla*	Manaus, AM, Brazil	Heartwood	HD	1-Nitro-2-phenylethane (46.0%), methyleugenol (52.9%), and linalool (5.0%)	[12]
*A. canelilla*	Manaus, AM, Brazil	Bark	HD	1-Nitro-2-phenylethane (72.6%) and methyleugenol (24.9%)	[12]
*A. canelilla*	Fatima de Chimanes, Bolivia	Bark	HD	1-Nitro-2-phenylethane (89.8%), methyleugenol (2.90%), and safrole (2.6%)	[83]
*A. canelilla*	Carajás National Forest, Marabá, PA, Brazil	Leaf	not reported	1-Nitro-2-phenylethane (95.3%) and limonene (1.2%)	[64]
*A. canelilla*	Carajás National Forest, Marabá, PA, Brazil	Bark	HD	1-Nitro-2-phenylethane (58.2%), methyleugenol (34.7%), and *epi*-α-cadinol (1.5%)	[64]
*A. canelilla*	Carajás National Forest, Marabá, PA, Brazil	Trunkwood	HD	1-Nitro-2-phenylethane (47.4%), *epi*-α-cadinol (19.9%), and methyleugenol (10.5%)	[64]
*A. canelilla*	Carajás National Forest, Marabá, PA, Brazil	Leaf	HD	1-Nitro-2-phenylethane (68.5%), linalool (8.8%), and spathulenol (4.8%)	[64]
*A. canelilla*	Carajás National Forest, Marabá, PA, Brazil	Bark	HD	1-Nitro-2-phenylethane (60.5%), methyleugenol (21.3%), and β-sesquiphellandrene (5.4%)	[64]
*A. canelilla*	Carajás National Forest, Marabá, PA, Brazil	Trunkwood	HD	1-Nitro-2-phenylethane (50.2%), methyleugenol (23.0%), and β-sesquiphellandrene (6.4%)	[64]
*A. canelilla*	Carajás National Forest, Marabá, PA, Brazil	Leaf	HD	Rainy season, Mn Mine: 1-nitro-2-phenylethane (70.6%) and methyleugenol (3.4%)	[84]
*A. canelilla*	Carajás National Forest, Marabá, PA, Brazil	Leaf	HD	Rainy season: Cu Mine: 1-nitro-2-phenylethane (94.5%) and methyleugenol (0.2%)	[84]
				Zoobotanic park: 1-nitro-2-phenylethane (95.3%) and methyleugenol (0.2%)	
*A. canelilla*	Carajás National Forest, Marabá, PA, Brazil	Leaf	HD	Dry season, Mn Mine: 1-nitro-2-phenylethane (39.0%) and methyleugenol (0.5%)	[84]
				Cu Mine: 1-nitro-2-phenylethane (39.3%) and methyleugenol (0.5%)	
				Zoobotanic park: 1-nitro-2-phenylethane (42.1%) and methyleugenol (0.6%)	
*A. canelilla*	Carajás National Forest, Marabá, PA, Brazil	Bark	HD	Rainy season, Mn Mine: 1-nitro-2-phenylethane (94.3%) and methyleugenol (1.0%)	[84]
				Cu Mine: 1-nitro-2-phenylethane (87.1%) and methyleugenol (8.7%)	
				Zoobotanic park: 1-nitro-2-phenylethane (78.2%) and methyleugenol (14.7%)	
*A. canelilla*	Carajás National Forest, Marabá, PA, Brazil	Bark	HD	Dry season: Mn Mine: 1-nitro-2-phenylethane (48.6%) and methyleugenol (45.3%)	[84]
				Cu Mine: 1-nitro-2-phenylethane (56.2%) and methyleugenol (39.5%)	
				Zoobotanic park: 1-nitro-2-phenylethane (68.1%) and methyleugenol (24.6%)	
*A. canelilla*	Carajás National Forest, Marabá, PA, Brazil	Trunkwood	HD	Rainy season, Mn Mine: 1-nitro-2-phenylethane (70.0%) and methyleugenol (17.7%)	[84]
*A. canelilla*	Carajás National Forest, Marabá, PA, Brazil	Trunkwood	HD	Rainy season, Cu Mine: 1-nitro-2-phenylethane (80.0%) and methyleugenol (10.7%)	[84]
				Zoobotanic park: 1-nitro-2-phenylethane (69.2%) and methyleugenol (15.3%)	
*A. canelilla*	Carajás National Forest, Marabá, PA, Brazil	Trunkwood	HD	Dry season, Mn Mine: 1-nitro-2-phenylethane (47.5%) and methyleugenol (48.5%)	[84]
				Cu Mine: 1-nitro-2-phenylethane (53.3%) and methyleugenol (38.0%)	
				Zoobotanic park: 1-nitro-2-phenylethane (73.3%) and methyleugenol (22.2%)	
*A. canelilla*	Cauaxi River, Paragominas, PA, Brazil	Bark	HD	1-Nitro-2-phenylethane (52.4%), methyleugenol (38.6%) and selin-11-en-4-α-ol (2.5%)	[84,85]
*A. canelilla*	Adolpho Ducke Forest Reserve, Manaus, AM, Brazil	Leaf	HD	1-Nitro-2-phenylethane (71.2%), benzaldehyde (4.8%), and β-selinene (4.5%)	[65]
*A. canelilla*	Adolpho Ducke Forest Reserve, Manaus, AM, Brazil	Thin twig	HD	1-Nitro-2-phenylethane (68.2%), eugenol (5.2%), and linalool (5.2%)	[65]
*A. canelilla*	Not reported	Leaf	SD	Commercial sample (market Ver-o-peso): 1-Nitro-2-phenylethane (99.1%) and eugenol (0.9%)	[77]
*A. canelilla*	Not reported	Wood	SD	Commercial sample: 1-Nitro-2-phenylethane (68.8%), methyleugenol (28.1%), and safrole (1.7%)	[77]
*A. canelilla*	Jatapu River, Novo Airão, AM, Brazil	Leaf and thin twig	HD	1-Nitro-2-phenylethane (91.8%), β-caryophyllene (1.6%), and selin-11-en-4-α-ol (1.3%)	[27]
*A. canelilla*	Jatapu River, Novo Airão, AM, Brazil	Trunkwood	HD	1-Nitro-2-phenylethane (92.1%), methyleugenol (4.30%), and eugenol (1.2%)	[27]
*A. canelilla*	Cauaxi River, Ulianópolis, PA, Brazil	Leaf and thin twig	HD	1-Nitro-2-phenylethane (74.0%), linalool (7.6%), and β-caryophyllene (3.5%)	[27]
*A. canelilla*	Cauaxi River, Ulianópolis, PA, Brazil	Barkwood	HD	1-Nitro-2-phenylethane (90.3%), selin-11-en-4-α-ol (3.5%), and methyleugenol (2.0%)	[27]
*A. canelilla*	Cauaxi River, Ulianópolis, PA, Brazil	Trunkwood	HD	1-Nitro-2-phenylethane (70.2%), methyleugenol (25.8%), and selin-11-en-4-α-ol (1.2%)	[27]
*A. canelilla*	Adolpho Ducke Forest Reserve, Manaus, AM, Brazil	Leaf	HD	Dry season: 1-nitro-2-phenylethane (88.9%), β-caryophyllene (4.21%), and β-phellandrene (0.80%)	[22]
*A. canelilla*	Adolpho Ducke Forest Reserve, Manaus, AM, Brazil	Leaf	HD	Rainy season: 1-nitro-2-phenylethane (88.5%),β-caryophyllene (5.04%), and β-phellandrene (1.14%)	[22]
*A. canelilla*	Itacoatiara, AM, Brazil	Leaf	HD	1-Nitro-2-phenylethane (52.2%), eugenol (8.71%) and methyleugenol (5.09%)	[102]
*A. canelilla*	Itacoatiara, AM, Brazil	Thin twig	HD	1-Nitro-2-phenylethane (92.7%), eugenol (0.3%) and methyleugenol (0.33%)	[102]
*A. canelilla*	Itacoatiara, AM, Brazil	Leaf	HD	Rainy season: 1-nitro-2-phenylethane (31.22-84.33%), β-caryophyllene (1.89-16.34%), and β-selinene (0.77-8.82%)	[101]
*A. canelilla*	Itacoatiara, AM, Brazil	Leaf	HD	Dry season: 1-nitro-2-phenylethane (13.17–74.55%), β-caryophyllene (4.19–12.64%), and β-selinene (1.09–14.98%)	[101]
*A. canelilla*	Itacoatiara, AM, Brazil	Stem	HD	Rainy season: 1-nitro-2-phenylethane (90.94–93.58%), benzeneacetaldehyde (0.48–1.98%) and eugenol (0.07–1.45%)	[101]
*A. canelilla*	Itacoatiara, AM, Brazil	Stem	HD	Dry season: 1-nitro-2-phenylethane (87.85–94.16%), linalool (0.05–3.08%), eugenol (0.08–1.19%)	[101]
*A. canelilla*	Itacoatiara, AM, Brazil	Trunkwood	HD	1-Nitro-2-phenylethane (83.68%), methyleugenol (14.83%) and *neo*-intermedeol (0.31%)	[86]
*A. canelilla*	Itacoatiara, AM, Brazil	Bark	HD	1-Nitro-2-phenylethane (83.2%), methyleugenol (16.2%), and benzaldehyde (0.4%)	[87]
*A. canelilla*	Adolpho Ducke Forest Reserve, Manaus, AM, Brazil	Leaf	HD	1-Nitro-2-phenylethane (87.34%), ethyl butanoate (3.10%), and α-humulene (0.68%)	[35]
*A. cinnamomiflora*	Los Andes, Merida, Venezuela	wood	HD	γ-Palmitolactone (54.0%), 1-*epi*-cubenol (9.56%), and δ-cadinene (6.05%)	[88]
*A. citrifolia*	Melgaço, PA, Brazil	Bark	HD	Safrole (16.7%), α-pinene (10.6%), and β-pinene (11.2%)	[73]
*A. duckei*	Manaus, AM, Brazil	Leaf	SD	Seasonal study, linalool (27.3–85.3%) and linalool oxides (5.3–19.7%)	[99]
*A. duckei*	Manaus, AM, Brazil	Thin twig	SD	Seasonal study, linalool (35.7–94.3%) and linalool oxides (3.0-18.7%)	[99]
*A. duckei*	Itacoatiara, AM, Brazil	Leaf and thin twig	HD	Linalool (81.8%), spathulenol (3.8%), and *cis*-linalool oxide (1.3%)	[51]
*A. duckei*	Adolpho Ducke Forest Reserve, Manaus, AM, Brazil	Leaf	HD	Rainy season: linalool (60.38%, 56.26%)	[103]
*A. duckei*	Adolpho Ducke Forest Reserve, Manaus, AM, Brazil	Leaf	HD	Dry season: linalool (62.40%, 76.69%)	[103]
*A. duckei*	Adolpho Ducke Forest Reserve, Manaus, AM, Brazil	Thin twig	HD	Linalool (89.34%), α-terpineol (3.06%), and *cis*-linalool oxide (1.94%)	[81]
*A. duckei*	Adolpho Ducke Forest Reserve, Manaus, AM, Brazil	Branch	HD	Linalool (93.60%), α-terpinolene (3.37%), and *cis*-linalool oxide (3.03%)	[29]
*A. gardneri*	Not reported	Leaf	HD	Benzyl benzoate (44.1%), cadinene (4.8%), and 1,8-cineole (3.6%)	[12]
*A. gardneri*	Not reported	Trunkwood	HD	Benzyl benzoate (78.1%) and phenylethyl benzoate (14.3%)	[12]
*A. guianensis*	Not reported	Leaf	HD	Benzyl benzoate (44.8%) and benzyl salicylate (16.7%)	[12]
*A. guianensis*	Not reported	Wood	HD	Benzyl benzoate (59.0%), benzyl salicylate (6.4%), and methylisoeugenol (5.0%)	[12]
*A. fragrans*	Curuá-una, PA, Brazil	Leaf and thin twig	HD	Linalool (32.4%), spathulenol (19.1%), and limonene (14.5%)	[51]
*A. hostmanniana*	Not reported	Bark	HD	(γ-asarone) 2,4,5-trimethoxyallylbenzene (94.5%)	[12]
*A. hostmanniana*	Not reported	Trunkwood	HD	(γ-asarone) 2,4,5-trimethoxyallylbenzene (98.6%)	[12]
*A. hostmanniana*	Forest of San Eusebio, Andrés Bello, Mérida, Venezuela	Leaf	HD	Benzyl benzoate (29.3%), δ-cadinene (12%), and β-caryophyllene (10.5%)	[68]
*A. panurensis*	Adolpho Ducke Forest Reserve, Manaus, AM, Brazil	Leaf	HD	β-Caryophyllene (33.5%), germacrene-D (25.4%), and α-copaene (7,5%)	[70]
*A. parviflora*	Zoobotanical Park of Emilio Goeldi Museum, Belém, PA, Brazil	Leaf	SD	Benzyl salicylate (34.3), benzyl benzoate (7.2%), and α-pinene (3.9%)	[106]
*A. parviflora*	Zoobotanical Park of Emilio Goeldi Museum, Belém, PA, Brazil	Root	SD	Benzyl benzoate (97.8%) and benzaldehyde (2.2%)	[106]
*A. parviflora*	Santarém, PA, Brazil	Leaf	HS-SPME	Linalool (29.6%), β-caryophyllene (10.9%), and α -phellandrene (10.5%)	[72]
*A. parviflora*	Tomé-Açu, PA, Brazil	Leaf	HD	Linalool (21.30%), β-phellandrene (21.06%), and α-phellandrene (7,25%)	[73]
*A. parviflora*	Tomé-Açu, PA, Brazil	Leaf	HD	β-Phellandrene (23,60%), linalool (12.64%), and spathulenol (4.73%)	[73]
*A. parviflora*	Belém, PA, Brazil	Leaf	HD	β-Phellandrene (15.1%), linalool (14.1%), and γ-eudesmol (12.9%)	[32]
*A. parviflora*	Belém, PA, Brazil	Branch	HD	γ-Eudesmol (16.8%), β-caryophyllene (15.7%), and linalool (12.4%)	[32]
*A. parviflora*	Santarém, PA, Brazil	Leaf	HD	Linalool (45.0%), β-phellandrene (17.3%), and α-phellandrene (4.1%)	[33,71]
*A. parviflora*	Santarém, PA, Brazil	Leaf	HD	Linalool (22.8%), β-caryophyllene (8.15%), and β-phellandrene (7.55%)	[34]
*A. parviflora*	Santarém, PA, Brazil	Thin wig	HD	Linalool (11.90%), aristolene (11.07%), and β-eudesmol (3.97%)	[34]
*A. parviflora*	Belém, PA, Brazil	Leaf	HD	Dry season: linalool (14,07%), spathulenol (7.0%), and oxygenated sesquiterpene (220) (6.7%)	[104]
*A. parviflora*	Belém, PA, Brazil	Leaf	HD	Rainy season: linalool (20.33%, 28.42%), *p*-cymene (13.04%, 17.54%), and α-phellandrene (12.74%, 14.87%)	[104]
*A. parviflora*	Adolpho Ducke Forest Reserve, Manaus, AM, Brazil	Leaf	HD	Linalool (40.02%), caryophyllene oxide (4.33%), 1,8-cineol (4.02%), and β-phellandrene (4.01%)	[35]
*A. puchury-minor*	Carajás National Forest, Marabá, PA, Brazil	Leaf	SD	Elemicin (23.46%), germacrene B (13.42%) and myristicin (8.43%)	[66]
*A. puchury-minor*	Carajás National Forest, Marabá, PA, Brazil	Stem	SD	(*E*)-Asarone (52.75%), methyleugenol (17.62%), and isolemicin (13.91%)	[66]
*A. puchury-minor*	Carajás National Forest, Marabá, PA, Brazil	Bark	SD	Methyleugenol (43.10%), (*E*)-asarone (29.95%), and isolemicin (11.87%)	[66]
*A. puchury-minor*	Carajás National Forest, Canaã dos Carajás, PA, Brazil	Leaf	HD	Elemicin (21.5%), bicyclogermacrene (15.4%), and spathulenol (11.3%)	[67]
*A. puchury-minor*	Carajás National Forest, Canaã dos Carajás, PA, Brazil	Bark	HD	(*E*)-Methylisoeugenol (43.1%), (*E*)-asarone (30.0%), (*Z*)-methylisoeugenol (9.0%)	[67]
*A. puchury-minor*	Carajás National Forest, Canaã dos Carajás, PA, Brazil	Wood	HD	(*E*)-Asarone (38.2%), (*E*)-isoelemicin (23.1%), (*Z*)-methylisoeugenol (14.8%)	[67]
*A. riparia*	Parintins, AM, Brazil	Leaf	HD	β-Caryophyllene (16.9%), α-humulene (14.9%) and bicyclogermacrene (14.1%)	[69]
*A. riparia*	Parintins, AM, Brazil	Branch	HD	(*E*)-Nerolidol (19.4%), elemol (16.2%), and α-humulene (10.9%)	[69]
*A. riparia*	Parintins, AM, Brazil	Barkwood	HD	Benzyl benzoate (30.9%), terpinen-4-ol (9.3%), and benzyl salicylate (7.9%)	[69]
*A. riparia*	Parintins, AM, Brazil	Trunkwood	HD	Benzyl benzoate (36.2%), benzyl salicylate (7.7%), and *cis*-calamenene (7.2%)	[69]
*A. rosaeodora*	not reported	Trunkwood	SD	Commercial samples: linalool (72.0-83.0%), *cis*-linalool oxide (1.10-5.80%), and *trans*-linalool oxide (1.10-5.20%)	[76]
*A. rosaeodora*	Curuá Una, Santarém, PA, Brazil	Leaf	SD	Linalool (73.0–78.0%), eremophyllene (4.50–6.0%), and cyclosatirene (1.40–2.70%)	[76]
				3*R*-(+)-linalool (77.8%)	
*A. rosaeodora*	Curuá Una, Santarém, PA, Brazil	Trunkwood	SD	Linalool (87.0–92.0%), *cis*-linalool oxide (1.0–1.68%), and *trans*-linalool oxide (0.90–1.60%)	[76]
				3*R*-(−)-linalool > 50.0%	
*A. rosaeodora*	Curuá Una, Santarém, PA, Brazil	Leaf	HD	Linalool (79.0%), oxygenated sesquiterpene (MW220) (5.4%), and β-selinene (2.0%)	[51]
*A. rosaeodora*	not reported	Not reported	HD	Commercial sample (*Institute for Medicinal Plants Research Dr*. *Josif Pancic*, Belgrade, Serbia): linalool (81.27%), α-terpineol (4.78%), *trans*-linalool oxide (2.10%)	[94]
*A. rosaeodora*	Not reported	Leaf	HD	Linalool (81.45%), β-selinene (1.65%), and α-selinene (1.39%)	[30]
				3*R*-(−)-linalool (29.3%) and 3*S*-(+)-linalool (70.7%)	
*A. rosaeodora*	Not reported	Trunkwood	HD	Linalool (85.0%), *trans*-linalool oxide (2.43%), and *cis*-linalool oxide (2.21%)	[30]
				3*R*-(−)-linalool (38.3%) and 3*S*-(+)-linalool (61.7%)	
*A. rosaeodora*	Presidente Figueiredo, AM, Brazil	Leaf	HS-SPME	Linalool (77.5%), *cis*-linalool oxide (7.7%), and *trans*-linalool oxide (5.6%)	[77]
*A. rosaeodora*	Presidente Figueiredo, AM, Brazil	Trunkwood	HS-SPME	Commercial sample: linalool (86.5%), *cis*-linalool oxide (4.9%), *trans*-linalool oxide (4.5%)	[77]
*A. rosaeodora*	Zoobotanical Park of Emilio Goeldi Museum, Belém, PA, Brazil	Leaf	HD	Seasonal study, linalool (68.0–96.1%)	[25]
*A. rosaeodora*	Zoobotanical Park of Emilio Goeldi Museum, Belém, PA, Brazil	Leaf	HD	Linalool (85.5%), β-selinene (0.9%), and *trans*-linalool oxide (0.8%)	[25]
*A. rosaeodora*	Zoobotanical Park of Emilio Goeldi Museum, Belém, PA, Brazil	Wood	HD	Linalool (84.8%), α-terpineol (2.9%), and geraniol (1.0%)	[25,91]
				3*S*-(−)-linalool (50.62%) and of 3*R*-(+)-linalool (49.38%)	[90]
*A. rosaeodora*	Novo Airão, AM, Brazil	Leaf	HD	Tachi morphological type: linalool (82.1%), spathulenol (2.0%), caryophyllene oxide (2.0%)	[25]
*A. rosaeodora*	Novo Airão, AM, Brazil	Trunkwood	HD	Tachi morphological type: linalool (84.8%), *cis*-linalool oxide (1.8%), and *trans*-linalool oxide (1.8%)	[25]
*A. rosaeodora*	Novo Airão, AM, Brazil	Leaf	HD	Preciosa morphological type: linalool (83.9%), caryophyllene oxide (2.0%), and *trans*-linalool oxide (1.7%)	[25]
*A. rosaeodora*	Novo Airão, AM, Brazil	Trunkwood	HD	Preciosa morphological type: linalool (79.7%), α-terpineol (5.6%), and *cis*-linalool oxide (2.7%)	[25]
*A. rosaeodora*	Novo Airão, AM, Brazil)	Leaf	HD	Itaúba morphological type: linalool (79.7%), caryophyllene oxide (3.2%), and β-selinene (1.6%)	[25]
*A. rosaeodora*	Novo Airão, AM, Brazil	Trunkwood	HD	Itaúba morphological type: linalool (78.9%), benzyl benzoate (2.7%), and β-selinene (1.0%)	[25]
*A. rosaeodora*	Novo Airão, AM, Brazil	Leaf	HD	Imbaúba morphological type: linalool (79.8%), spathulenol (4.0%), and *trans*-linalool oxide (1.7%)	[25]
*A. rosaeodora*	Novo Airão, AM, Brazil	Trunkwood	HD	Imbaúba morphological type: linalool (76.9%), α-terpineol (3.8%), and *cis*-linalool oxide (1.6%)	[25]
*A. rosaeodora*	French Guiana	Leaf	HD	3*S*-(+)-linalool (78–89%)	[107]
*A. rosaeodora*	French Guiana	Branch	HD	3*S*-(+)-linalool (5–28%)	[107]
*A. rosaeodora*	French Guiana	Trunkwood	HD	3*R*-(−)-linalool (95–99%)	[107]
*A. rosaeodora*	French Guiana	Trunkwood	HD	3*S*-(+)-linalool (73.0%), α-terpineol (18.8%), nerol (1.7%)	[107]
*A. rosaeodora*	Novo Airão, AM, Brazil	Trunkwood	HD	Linalool (87.7%), α-terpineol (3.1%), *trans*-linalool oxide (1.5%), and *cis*-linalool oxide (1.5%)	[92]
*A. rosaeodora*	Adolpho Ducke Forest Reserve, Manaus, AM, Brazil	Stem	HD	Linalool (86.0%), caryophyllene oxide (2.8%), and *trans*-linalool oxide (1.5%)	[70]
*A. rosaeodora*	not reported	Not reported	SD	Commercial sample (Erbamea-Istrana, Treviso, Italy): linalool (60.1%), limonene (19.2%), and geraniol (7.8%)	[95]
*A. rosaeodora*	not reported	Not reported	SD	Commercial sample: linalool (80%), and α-terpineol (4.5%)	[97]
*A. rosaeodora*	Maués, AM, Brazil	Leaf and thin twig	SD	A four years sample: linalool (82.15%), α-terpineol (3.60%), and geraniol (1.33%)	[78,79]
*A. rosaeodora*	Maués, AM, Brazil	Leaf and thin twig	SD	A ten years sample: linalool (90.5%), α-terpineol (1.11%), and α-selinene (0.73%)	[79]
*A. rosaeodora*	Maués, AM, Brazil	Leaf and thin twig	SD	A twenty years sample: linalool (87.1%), benzyl benzoate (1.61%), and α-terpineol (1.21%)	[79]
*A. rosaeodora*	Tomé-Açu, PA, Brazil	Leaf	HD	Linalool (50.81%), spathulenol (4.46%), and β-selinene (4.35%)	[73]
*A. rosaeodora*	Tomé-Açu, PA, Brazil	Leaf	HD	Linalool (55.91%), *cis*-linalool furanoxide (5.59%), and *trans*-linalool furanoxide (4.43%)	[73]
*A. rosaeodora*	Belém, PA, Brazil	Leaf	HD	Linalool (51.75%), spathulenol (3.82%), and β-selinene (3.79%)	[73]
*A. rosaeodora*	Belém, PA, Brazil	Leaf	HD	Linalool (57.17%), *cis*-linalool furanoid (4.21%), spathulenol (3.92%)	[73]
*A. rosaeodora*	Belém, PA, Brazil	Leaf	HD	Linalool (43.02%), β-selinene (5.84%), and spathulenol (4.46%)	[73]
*A. rosaeodora*	Belém, PA, Brazil	Leaf	HD	Linalool (42.61%), β-selinene (5.23%), and α-selinene (4.26%)	[73]
*A. rosaeodora*	Belém, PA, Brazil	Leaf	HD	Linalool (45.28%), β-selinene (5.11%), and α-selinene (4.11%)	[73]
*A. rosaeodora*	Belém, PA, Brazil	Leaf	HD	Linalool (42.67%), spathulenol (7.20%), and β-selinene (4.88%)	[73]
*A. rosaeodora*	Belém, PA, Brazil	Leaf	HD	Linalool (52.22%), β-selinene (4.24%), and α-selinene (3.53%)	[73]
*A. rosaeodora*	Belém, PA, Brazil	Leaf	HD	Linalool (55.10%), *cis*-linalool furanoid (4.28%), and spathulenol (4.25%)	[73]
*A. rosaeodora*	Belém, PA, Brazil	Leaf	HD	Linalool (43.96%), β-selinene (5.25%), and α-selinene (4.24%)	[73]
*A. rosaeodora*	Belém, PA, Brazil	Leaf	HD	Linalool (44.66%), β-selinene (5.40%), and α-selinene (4.49%)	[73]
*A. rosaeodora*	Belém, PA, Brazil	Leaf	HD	Linalool (56.29%), *cis*-linalool furanoid (4.79%), and spathulenol (3.87%)	[73]
*A. rosaeodora*	Belém, PA, Brazil	Leaf	HD	Linalool (52.72%), spathulenol (5.12%), and β-selinene (4.0%)	[73]
*A. rosaeodora*	Belém, PA, Brazil	Leaf	HD	Linalool (46.90%), β-selinene (4.92%), and spathulenol (4.53%)	[73]
*A. rosaeodora*	Belém, PA, Brazil	Leaf	HD	Linalool (49.24%), spathulenol (6.47%), and β-selinene (4.09%)	[73]
*A. rosaeodora*	Belém, PA, Brazil	Leaf	HD	Linalool (38.48%), β-selinene (6.41%), and α-selinene (5.58%)	[73]
*A. rosaeodora*	Belém, PA, Brazil	Leaf	HD	Linalool (47.49%), benzyl benzoate (6.25%), and β-selinene (4.02%)	[73]
*A. rosaeodora*	Belém, PA, Brazil	Leaf	HD	Linalool (45.41%), β-selinene (4.31%), and spathulenol (4.70%)	[73]
*A. rosaeodora*	Belém, PA, Brazil	Leaf	HD	Linalool (45.99%), α-copaene (5.06%), and β-selinene (4.04%)	[73]
*A. rosaeodora*	Belém, PA, Brazil	Leaf	HD	Linalool (57.22%), spathulenol (6.53%), and α-copaene (5.48%)	[73]
*A. rosaeodora*	Belém, PA, Brazil	Leaf	HD	Linalool (64.26%), α-copaene (3.27%), and spathulenol (3.26%)	[73]
*A. rosaeodora*	Belém, PA, Brazil	Leaf	HD	Linalool (52.68%), spathulenol (4.56%), and β-selinene (4.19%)	[73]
*A. rosaeodora*	Belém, PA, Brazil	Leaf	HD	Linalool (49.93%), spathulenol (4.44%), and β-selinene (3.89%)	[73]
*A. rosaeodora*	Belém, PA, Brazil	Leaf	HD	Linalool (51.88%), β-selinene (4.12%), and *cis*-linalool furanoid (3.18%)	[73]
*A. rosaeodora*	Belém, PA, Brazil	Leaf	HD	Linalool (54.36%), β-selinene (4.13%), and α-copaene (3.95%)	[73]
*A. rosaeodora*	Belém, PA, Brazil	Leaf	HD	Linalool (71.05%), spathulenol (3.73%), and α-copaene (2.90%)	[73]
*A. rosaeodora*	Belém, PA, Brazil	Leaf	HD	Linalool (70.3%), spathulenol (6.20%), and α-copaene (2.07%)	[73]
*A. rosaeodora*	Belém, PA, Brazil	Leaf	HD	Linalool (43.66%), β-selinene (6.29%), and spathulenol (6.18%)	[73]
*A. rosaeodora*	Belém, PA, Brazil	Leaf	HD	Linalool (50.35%), β-selinene (4.22%), and α-selinene (3.50%)	[73]
*A. rosaeodora*	Belém, PA, Brazil	Leaf	HD	Linalool (48.39%), α-copaene (3.73%), and β-selinene (3.55%)	[73]
*A. rosaeodora*	Belém, PA, Brazil	Leaf	HD	Linalool (61.96%), spathulenol (3.75%), and β-selinene (3.10%)	[73]
*A. rosaeodora*	Curuá-una, Santarém, PA, Brazil	Leaf	HD	Linalool (55.09%), β-selinene (4.49%), and α-selinene (4.14%)	[73]
*A. rosaeodora*	Curuá-una, Santarém, PA, Brazil	Leaf	HD	Linalool (55.93%), β-selinene (3.79%), and α-selinene (3.18%)	[73]
*A. rosaeodora*	Curuá-una, Santarém, PA, Brazil	Leaf	HD	Linalool (48.10%), β-selinene (4.81%), and spathulenol (4.62%)	[73]
*A. rosaeodora*	Santarém, PA, Brazil	Leaf	HD	Linalool (88.6%), *cis*-linalool oxide (1.5%), and (*E*)-nerolidyl acetate (1.5%)	[33,71]
*A. rosaeodora*	Brazil	Trunkwood	SD	Commercial sample (Oshadhi Ltd. (Cambridge, England): linalool (44.4%), linalyl acetate (31.4%), and β-caryophyllene (10.5%)	[96]
*A. rosaeodora*	not reported	Trunkwood	SD	Commercial sample: linalool (86.23%), *cis*-linalool oxide (1.06%), and β-selinene (0.95%)	[98]
*A. rosaeodora*	Maués, AM, Brazil	Leaf	HD	Linalool (81.32%, 83.59%), spathulenol (2.52%, 1.98%), β-selinene (1.4%, 1.4%), and camphene (0.04%, 0.30%)	[80]
*A. rosaeodora*	Maués, AM, Brazil	Branch	HD	Linalool (83.88%, 79.49%), β-selinene (0.38%, 0.63%), spathulenol (0.37%, 1.04%), and camphene (0.17%, 1.03%)	[80]
*A. rosaeodora*	Maués, AM, Brazil	Leaf and branch	HD	Linalool (82.11%, 81.33%), spathulenol (1.01%, 2.06%), β-selinene (0.64%, 1.15%), camphene (0.04%, 0.71%)	[80]
*A. rosaeodora*	Maués, AM, Brazil	Stem	HD	Linalool (86.12%), β-selinene (0.43%), camphene (0.11%), and spathulenol (0.11%)	[80]
*A. rosaeodora*	Novo Aripuanã, AM, Brazil	Leaf	HD	Linalool (71.76%, 73.89%), spathulenol (3.16%, 2.13%), β-selinene (2.71%, 2.19%), and camphene (0.76%)	[80]
*A. rosaeodora*	Novo Aripuanã, AM, Brazil	Branch	HD	Linalool (81.53%, 65.08%), β-selinene (0.61%, 0.76%), camphene (0.29%, 1.62%), and spathulenol (0.34%, 0.93%)	[80]
*A. rosaeodora*	Novo Aripuanã, AM, Brazil	Leaf and branch	HD	Linalool (75.57%, 73.24%), spathulenol (2.03%, 2.15%), β-selinene (1.62%, 1.79%), and camphene (0.97%)	[80]
*A. rosaeodora*	Novo Aripuanã, AM, Brazil	Stem	HD	Linalool (81.77%), β-selinene (0.64%), spathulenol (0.24%), and camphene (0.16%)	[80]
*A. rosaeodora*	Manaus, AM, Brazil	Leaf	HD	Dry season: linalool (69.0%), β-phellandrene (2.9%), and bulnesol (1.8%)	[28]
*A. rosaeodora*	Manaus, AM, Brazil	Leaf	HD	Rainy season: linalool (71.0%), butanoic acid (3.5%), and *trans*-linalool oxide (2.8%)	[28]
*A. rosaeodora*	Manaus, AM, Brazil	Thin twig	HD	Dry season: linalool (78.0%), β-phellandrene (1.5%), and α-eudesmol (1.4%)	[28]
*A. rosaeodora*	Manaus, AM, Brazil	Thin twig	HD	Rainy season: linalool (84.0%), butanoic acid (3.0%), and *cis*-linalool oxide (2.0%)	[28]
*A. rosaeodora*	Floresta Nacional do Tapajós- Rurópolis (PA, Brazil)	Leaf and thin twig	HD	Linalool (83.7%), aromadendrene oxide (2.5%), and spathulenol (1.6%)	[74]
*A. rosaeodora*	Reserva Extrativista Tapajós-Arapiuns (PA, Brazil)	Leaf and thin twig	HD	Linalool (39.6%), α-phellandrene (22.8%) and *p*-cymene (7.0%)	[74]
*A. rosaeodora*	Adolpho Ducke Forest Reserve, Manaus, AM, Brazil	Leaf and thin twig	HD	Linalool (93.60%), α-terpinolene (3.37%), and *cis*-linalool oxide (3.03%)	[81,82]
*A. rosaeodora*	Adolpho Ducke Forest Reserve, Manaus, AM, Brazil	Stem	HD	Linalool (63.16%), *trans*-linalool oxide (9.73%) and *cis*-linalool oxide (7.69%)	[93]
*A. terminalis*	Zoobotanical Park of Emilio Goeldi Museum, Belém, PA, Brazil	Leaf and thin twig	HD	α-Phellandrene (32.8%), linalool (21.7%), and *p*-cymene (16.7%)	[75]
*A. terminalis*	Zoobotanical Park of Emilio Goeldi Museum, Belém, PA, Brazil	Inflorescence	HD	Linalool (36.2%), α-phellandrene (30.7%), and (*E*)-β-ocimene (22.3%)	[75]

HD: Hydrodistillation; SD: Steam Distillation; HS-SPME: Headspace-Solid Phase Micro-Extraction.

## 7. Biological Activities 

**Figure 6:**
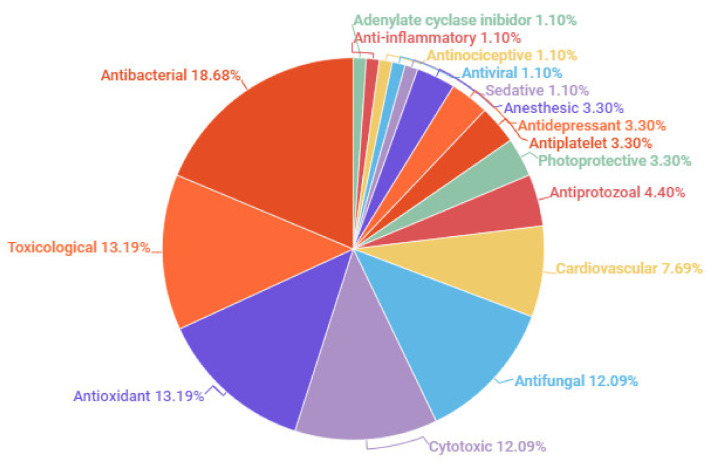
Studies reported on biological activities of essential oil of *Aniba* species from the Amazon.

The studies on biological activities of EOs of *Aniba* species from the Amazon correspond to 63 oil samples. Among them, six samples had no chemical composition analysis. Several oils presented more than one specific activity, and the most frequent were antibacterial, toxicological, antifungal, antioxidant, and cytotoxic activities. The percentages of biological activities report the essential oils of *Aniba* species from the Amazon, and their details of biological assays are presented in Figure 6 and Table 2.

### 7.1. Antibacterial Activity

Antibacterial properties of various *Aniba* essential oils were evaluated using the disk diffusion and plate microdilution bioassay. 

The oil of *A. canelilla* trunkwood was tested against the human pathogenic bacteria *Staphylococcus aureus* and *S. homini*. Oil (50 mg/mL) containing 1-nitro-2-phenylethane (73.0%) and methyleugenol (19.2%) was active against *S. aureus* (S-methicillin sensitive and R-methicillin resistant) with a zone of inhibition of 12 mm and 15 mm, respectively [77]. According to [108], inhibition zones diameter can be scored as weak (10–13.9 mm), moderate (14–18 mm), or strong (>18 mm).

The leaf oil of *A. parviflora*, composed of linalool (45.0%), β-phellandrene (17.3%) and α-phellandrene (4.1%), and the leaf oil of *Aniba rosaeodora*, containing linalool (88.6%), collected in Santarém (PA, Brazil), showed effective antibacterial activity against *Escherichia coli*, *Klebsiella pneumoniae*, *Staphylococcus aureus*, *S. epidermidis Enterococcus faecalis*, and *Streptococcus pyogenes* (MIC 1.3–10 μL/mL), and the antibiotics ampicillin and gentamicin (10 µg/disk) were applied as the reference standard [33]. Usually, the antimicrobial activity of essential oil is classified according to MIC values as strong (MIC from 50 to 500 µg/mL), moderate (MIC from 600 to 1500 μg/mL), and weak (MIC > 1500 μg/mL) [109]. Essential oils of leaves and branches from *A. parviflora*, also occurring at Santarém, PA, Brazil, containing linalool (22.8%), β-caryophyllene (8.15%), and β-phellandrene (7.55%) in the leaves, and linalool (11.90%), aristolene (11.7%) and β-eudesmol (3.97%) in the branches were tested against *S. aureus*, *E. faecalis*, *E. coli*, and *Pseudomonas aeruginosa*. Both oils exhibited activity against *S. aureus* and *E. faecalis* (MIC, 2000 μg/mL), considered weak or inactive. Gentamicin (10 μg) was used as positive control [34].

Essential oils have been used in diets for chickens as alternative antibiotic products and growth promoters. Due to their antimicrobial properties, the trunkwood oil of *A. rosaeodora*, collected in Belém (PA, Brazil), was evaluated in vivo against *E. coli* from the gastrointestinal tract broiler of chickens. Linalool (84.8%), α-terpineol (2.9%), and geraniol (1.0%) were the main compounds in the tested oil. Broilers were fed with rosewood oil at 40 days of age, and samples from the gastrointestinal tracts were inoculated on plates. The rosewood oil was also evaluated as a growth promoter but did not influence broilers’ growth or fattening performance. The oil at 0.45% reduced the relative weight of the intestines. The commercial growth promoter virginiamycin (100 ppm) was used as control [110].

The oil of leaves and thin branches from *A. rosaeodora*, sampled in Adolpho Ducke Forest Reserve (AM, Brazil), containing linalool (93.6%), α-terpinolene (3.3%), and *cis*-linalool oxide (3.0%) was evaluated by disk-diffusion method against bacteria isolated from a marine environment. The MIC of *A. rosaeodora* oil ranged from 250 to 450 μg/mL, compared to standard linalool (550–650 μg/mL), and the antibiotics amoxicillin (8–16 μg/mL), gentamycin (2–8 μg/mL), and polymyxin B (16 μg/mL). *Aniba rosaeodora* oil was more efficient against *Aeromonas caviae* and *Enterococcus faecalis* than the standard linalool. Linalool exhibited more significant activity against *Klebsiella pneumonia* and *Providencia stuartii* compared to the oil, while the oil and linalool presented the same activity against *Aeromonas hydrophila* [81]. The oil from stems of *A. rosaeodora* was also tested against *E. coli* and *S. aureus* and presented MIC of 200 and 150 μg/mL, respectively. The inhibition halos ranging from 11 to 15 mm, and the minimum bactericidal concentration (MBC) ranging from 400–350 μg/mL [82].

Antibacterial properties of other *Aniba* oils were also evaluated using the microdilution method. The leaf oil of *A. hostmanniana*, dominated by benzyl benzoate (29.3%), δ-cadinene (12.0%), and β-caryophyllene (10.5%), was tested against the bacteria *E. coli*, *P. aeruginosa*, and *K. pneumoniae* (Gram-negative), and *S. aureus* (Gram-positive). Oil displayed significant MIC values to *P. aeruginosa* (900 μg/mL), *S. aureus* (900 μg/mL), and *K. pneumoniae* (1250 μg/mL), while to *E. coli*, the oil did not display sensitivity [68]. The essential oils of *A. parviflora* from Belém (PA, Brazil), containing β-phellandrene (15.1%), linalool (14.1%), and γ-eudesmol (12.9%) in the leaves, and γ-eudesmol (16.8%), β-caryophyllene (15.4%), and linalool (12.4%) in the branches, showed potent activity against *E. coli* (MIC: 19.5 μg/mL) and moderate activity against *S. aureus* (MIC: 625.0 μg/mL), *S. epidermidis* (625.0–1250.0 μg/mL) and *P. aeruginosa* (1250.0 μg/mL). On the other hand, for *Bacillus cereus*, the same leaf oil displayed a variable activity (MIC: 312.5–1250.0 μg/mL). Gentamicin (19.5 μg/mL) was used as control [32].

**Table 2:** Main constituents and biological activities of essential oils of *Aniba* species from the Amazon.

*Aniba* Species	Collection Site	Plant Part	Major Componentes	Bioactivities	References
*A. canelilla*	Adolpho Ducke Forest Reserve, Manaus, AM, Brazil	Leaf	1-Nitro-2-phenylethane (88.9%), β-caryophyllene (4.21%), and β-phellandrene (0.80%)	Anti-leishmanial (IC_50_ 40 μg/mL, *Leishmania amazonensis* promastigotes); cytotoxic (mice BALB-c macrophage, MTT assay, IC_50_ 9.3 μg/mL); toxicological (LC_50_ 68.37 μg/mL, *Artemia salina* lethality)	[22]
*A. canelilla*	Adolpho Ducke Forest Reserve, Manaus, AM, Brazil	Leaf	1-Nitro-2-phenylethane (88.9%), β-caryophyllene (4.21%), and β-phellandrene (0.80%)	Anti-leishmanial (IC_50_ 40 μg/mL, *Leishmania amazonensis* promastigotes); cytotoxic (mice BALB-c macrophage, MTT assay, IC_50_ 9.3 μg/mL); toxicological (LC_50_ 68.37 μg/mL, *Artemia salina* lethality)	[22]
*A. canelilla*	Cauaxi River, Paragominas, PA, Brazil	Bark	1-Nitro-2-phenylethane (52.4%), methyleugenol (38.6%) and selin-11-en-4-α-ol (2.5%);	Cardiovascular, hypotension and bradycardia, EO at 1–10 mg/kg; vasorelaxant effects, IC_50_ 19 μg/mL;	[23]
*A. canelilla*	Cauaxi River, Paragominas, PA, Brazil	Bark	1-Nitro-2-phenylethane, isolated	Cardiovascular, hypotension and bradycardia, EO at 1–20 mg/kg; vasorelaxant effects, IC_50_ 29.6 μg/mL	[23]
*A. canelilla*	Cauaxi River, Paragominas, PA, Brazil	Bark	1-Nitro-2-phenylethane (52.4%), methyleugenol (38.6%) and selin-11-en-4α-ol (2.5%), and 1-nitro-2-phenylethane, isolated	Cardiovascular, vasorelaxant effects (IC_50_ 294.19 μg/mL) and 1-nitro-2-phenylethane isolated (IC_50_ 501.27 μg/mL)	[24]
*A. canelilla*	Jatapu River, Novo Airão, AM, Brazil	Trunk wood	1-Nitro-2-phenylethane (92.1%), methyleugenol (4.30%), and eugenol (1.2%)	Antioxidant (EC_50_ 223.81 μg/mL, DPPH method); toxicological (LC_50_ 21.61 μg/mL, *Artemia salina* lethality)	[27]
*A. canelilla*	Cauaxi River, Ulianópolis, PA, Brazil	Trunk wood	1-Nitro-2-phenylethane (70.2%), methyleugenol (25.8%), and selin-11-en-4α-ol (1.2%)	Antioxidant (EC_50_ 172.52 μg/mL, DPPH method); toxicological (LC_50_ 21.61 μg/mL, *Artemia salina* lethality assay)	[27]
*A. canelilla*	Adolpho Ducke Forest Reserve, Manaus, AM, Brazil	Leaf	1-Nitro-2-phenylethane (87.34%), ethyl butanoate (3.10%), and α-humulene (0.68%)	Antifungal (*Aspergillus flavus*, *Colletotrichum guaranicola*, MIC 0.15 μg/mL; *A. niger*, MIC 0.3 μg/mL; *Fusarium oxysporum*, *F. solani*, *C. gloeosporioides, C. musae,* MIC 0.62 μg/mL; *Alternaria alternata* MIC 5.0 μg/mL, agar-well diffusion method)	[35]
*A. canelilla*	not reported	Bark stem	1-Nitro-2-phenylethane (73.0%), methyleugenol (19.2%), safrole (3.7%), and eugenol (1.5%)	Antimicrobial (*Staphylococcus aureus*, *Candida albicans*, *C. parapsilosis* and *C. krusei*, agar diffusion method)	[77]
*A. canelilla*	Cauaxi River, Paragominas, PA, Brazil	Bark	1-Nitro-2-phenylethane (52.4%), methyleugenol (38.6%) and selin-11-en-4α-ol (2.5%)	Cardiovascular (*Rattus norvegicus* male Wistar rat model), hypotension and bradycardia EO at 1, 5, 10, and 20 mg/kg; vasorelaxant effects, IC_50_ 109.5 μg/mL	[85]
*A. canelilla*	Itacoatiara, AM, Brazil	Wood	1-Nitro-2-phenylethane (83.68%), methyleugenol (14.83%) and neointermedeol (0.31%)	Trypanocide (*Trypanosoma evansi*, mortality after 6 h, EO at 0.5 to 2.0%); Cytotoxic (human lymphocytes, EO at 0.5 to 2.0%, MTT assay)	[86]
*A. canelilla*	Cauaxi River, Ulianópolis, PA, Brazil	Bark wood	1-Nitro-2-phenylethane	Antinociceptive and anti-inflammatory (abdominal writhing method)	[110]
*A. canelilla*	Cauaxi River, Ulianópolis, PA, Brazil	Trunk wood	1-Nitro-2-phenylethane, isolated	Anti-inflammatory (25 and 50 mg/kg, paw and ear edema in male Swiss mice and Wistar rats)	[111]
*A. canelilla*	Adolpho Ducke Forest Reserve, Manaus, AM, Brazil.	Leaf	Not reported	Photoprotective (dry season, FPS 7.54; rainy season FPS 14.08, spectrophotometric method)	[112]
*A. canelilla*	Adolpho Ducke Forest Reserve, Manaus, AM, Brazil.	Thin twig	Not reported	Photoprotective (dry season, FPS 5.49; rainy season FPS 6.93, spectrophotometric method)	[112]
*A. canelilla*	Cauaxi River, Paragominas, PA, Brazil	Bark	1-Nitro-2-phenylethane, isolated	Cardiovascular (male Wistar rat model), induced hypotensive and bradycardic, EO at 1–10 mg/kg; vasorelaxant effects, IC_50_ 60.1 μg/mL	[113]
*A. canelilla*	Cauaxi River, Paragominas, PA, Brazil	Bark	1-Nitro-2-phenylethane, isolated	Cardiovascular (Male Wistar rat model); vasorelaxant effects IC_50_ 203.1 μM, contractions induced by phenylephrine; Hyp9 IC_50_ 119.0 μM and phorbol 12,13-dibutyrate IC_50_ 43 μM	[114]
*A. canelilla*	not reported	Not reported	1-Nitro-2-phenylethane, isolated	Cardiovascular, vasorelaxant effects at concentration of 0.1–100 μg/mL	[115]
*A. canelilla*	Cauaxi River, Ulianópolis, PA, Brazil	Trunk wood	1-Nitro-2-phenylethane (70.2%), methyleugenol (25.8%), and selin-11-en-4α-ol (1.2%); 1-nitro-2-phenylethane, isolated	Acetylcholinesterase (detection limit of 0.01 ng to EO and 1-nitro-2-phenylethane, isolated, TLC bioautography method)	[116]
*A. duckei*	Adolpho Ducke Forest Reserve, Manaus, AM, Brazil	Twig	Linalool (93.6%), α-terpinolene (3.37%), and *cis*-linalool oxide (3.03%)	Antifungal (*Colletotrichum gloesporioides* and *Fusarium oxysporum*, mycelial growth inhibition in 100% at 0.4%)	[29]
*A. duckei*	Adolpho Ducke Forest Reserve, Manaus, AM, Brazil	Twig	Linalool (89.34%), α-terpineol (3.06%), and *cis*-linalool oxide (1.94%)	Toxicological (*Aedes aegypti*, LC_50_ 250.61 μg/mL, EO; LC_50_ 279.89 μg/mL, 3*R*-(–)-linalool; LC_50_ 346 μg/mL, (±)-linalool	[81]
			3*R*-(–)-linalool and (±)-linalool standard		
*A. duckei*	Adolpho Ducke Forest Reserve, Manaus, AM, Brazil	Leaf	Not reported	Toxicological (*Artemia franciscana*, 100% of larval mortality after 10min, EO at 2 μg/mL)	[117]
*A. duckei*	Adolpho Ducke Forest Reserve, Manaus, AM, Brazil	Twig	Not reported	Toxicological (*Aedes aegypti*, LC_90_ 54 × 10^3^ μg/mL and LC_50_ 2.2 × 10^2^ μg/mL)	[117]
*A. hostmanniana*	Forest of San Eusebio, Andrés Bello, Mérida, Venezuela	Leaf	Benzyl benzoate (29.3%), δ-cadinene (12%), and β-caryophyllene (10.5%)	Antibacterial (*Pseudomonas aeruginosa* and *Staphylococcus aureus,* MIC 900 μg/mL, *Klebsiella pneumonia,* MIC1250 μg/mL, broth microdilution method)	[68]
*A. panurensis*	Adolpho Ducke Forest Reserve, Manaus, AM, Brazil	Leaf	β-Caryophyllene (33.5%), germacrene-D (25.4%), α-copaene (7.5%) and β-bourbonene (7.1%)	Antioxidant (EC_50_ > 1000 μg/mL, DPPH method); Antiplatelet activity (3.57%)	[70]
*A. parviflora*	Belém (PA, Brazil)	Leaf	β-Phellandrene (15.1%), linalool (14.1%), and γ-eudesmol (12.9%)	Antibacterial (*Escherichia coli*, MIC 19.5 μg/mL; *Bacillus cereus*, MIC: 312.5 μg/mL; *Staphylococcus aureus*, *S. epidermidis*, MIC: 625 μg/mL, *Pseudomonas aeruginosa*, MIC 1250 μg/mL, microdilution method); antioxidant (90.1–287.9 mg TE/mL, DPPH method); cytotoxic (MCF-7 mammary adenocarcinoma, IC_50_ 67.9 μg/mL, MTT assay)	[32]
*A. parviflora*	Belém (PA, Brazil)	Branch	γ-Eudesmol (16.8%), β-caryophyllene (15.7%), and linalool (12.4%)	Antibacterial (*Escherichia coli*, MIC 19.5 μg/mL; *Bacillus cereus*, MIC: 1250 μg/mL; *Staphylococcus aureus* MIC: 625 μg/mL, *S. epidermidis* 1250 μg/mL, *Pseudomonas aeruginosa*, MIC 1250 μg/mL, microdilution method); antioxidant (94.1–358.4 mgTE/mL, DPPH method); cytotoxic (MCF-7 mammary adenocarcinoma, IC_50_ 102.2 μg/mL MTT assay)	[32]
*A. parviflora*	Curuá-una, Santarém, PA, Brazil	Leaf	Linalool (45.0%), β-phellandrene (17.3%), and α-phellandrene (4.1%)	Antibacterial (*Klesbsiella pneumoniae Enterococcus faecalis*, *Staphylococcus aureus* and *S. epidermidis* MIC > 10 μL/mL, *Streptococcus pyogenes* MIC 1.3 μL/mL, agar disk diffusion method)	[33]
*A. parviflora*	Santarém, PA, Brazil	Leaf	Linalool (22.8%), caryophyllene (8.15 %), β-phellandrene (7.55%), and *o*-cymene (6.19%)	Antibacterial (*Staphylococcus aureus* and *Enterococcus faecalis*, MIC 2,0 mg/mL, agar disk diffusion method)	[34]
		Twig	Linalool (11.90%), aristolene (11.7%),β-eudesmol (3.97%), and spathulenol (3.51%)		
*A. parviflora*	Adolpho Ducke Forest Reserve, Manaus, AM, Brazil	Leaf	Linalool (40.02%), caryophyllene oxide (4.33%), 1,8-cineole (4.02%), and β-phellandrene (4.01%)	Antifungal (*Aspergillus flavus*, *Fusarium solani*, *F. oxysporum,* MIC 0.62 µL/mL; *A. niger*, *Colletotrichum guaranicola,* MIC 2.5 µL/mL; *C. gloeosporioides,* MIC 0.62 µL/mL; *Alternaria alternata, C. musae,* MIC 5.0 µL/mL, agar-well diffusion method)	[35]
*A. parviflora*	Curuá-una, Santarém, PA, Brazil	Leaf	Linalool (45.0%), β-phellandrene (17.3%), α-phellandrene (4.1%), and (*E*)-caryophyllene (3.9%)	Antidepressant activity (Male Wistar rats, EO at 85 mg/kg)	[71]
*A. parviflora*	Santarém, PA, Brazil	Leaf	Linalool (29.60%), β-caryophyllene (10.9%), and α-phellandrene (10.5%)	Anesthetic potential to the fish species *Colossoma macropomum*. EO at 0.1 μL/mL light sedation (46.4 s), deep sedation (120.8 s), deep anesthesia (333.2 s)	[118]
*A. parviflora*	Curauá, Santarém, PA, Brazil	Bark	Linalool (16.3%), α-humulene (14.5%), δ-cadinene (10.2%), α-copaene (9.51%) and germacrene B (7.58%)	Cytotoxic (Human hepatocellular carcinoma cells HepG2; IC_50_ values of 9.05 μg/mL; xenograft model).	[119]
*A. rosaeodora*	Manaus, AM, Brazil	Leaf	Dry season: linalool (69.0%), β-phellandrene (2.9%), and bulnesol (1.8%)	Antifungal activity (*Colletotrichum gloeosporioides*, MIC 1.25 μL/mL, *Colletotrichum* sp., MIC 2.5 μL/mL; *C. guaranicola*, MIC 0.62 μL/mL; *Alternaria alternata*, MIC 1.25 μL/mL, agar-well diffusion method)	[28]
			Rainy season: linalool (71.0%), butanoic acid (3.5%), and *trans*-linalool oxide (2.8%)	Antifungal activity (*Colletotrichum gloeosporioides* rainy season, MIC 2.5 μL/mL; *Colletotrichum* sp., MIC 5.0 μL/mL; *C. guaranicola*, MIC 1.25 μL/mL; *Alternaria alternata*, MIC 2.5 μL/mL, agar well diffusion method)	
*A. rosaeodora*	Manaus, AM, Brazil	Thin twig	Dry season: linalool (78.0%), β-phellandrene (1.5%), and α-eudesmol (1.4%)	Antifungal activity (*Colletotrichum gloeosporioides*, MIC 1.25 μL/mL; *Colletotrichum* sp., dry season, MIC 2.5 μL/mL; *C. guaranicola*, MIC 1.25 μL/mL; *Alternaria alternata*, MIC 1.25 μL/mL, agar well diffusion method)	[28]
			Rainy season: linalool (84.0%), butanoic acid (3.0%), and *cis*-linalool oxide (2.0%)	Antifungal activity (*Colletotrichum gloeosporioides*, MIC 2.5 μL/mL; *Colletotrichum* sp., MIC 5.0 μL/mL; *C. guaranicola*, MIC 1.25 μL/mL; *Alternaria alternata*, MIC 2.5 μL/mL, agar well diffusion method)	
*A. rosaeodora*	Curuá-una, Santarém, PA, Brazil	Leaf	Linalool (88.6%), *cis*-linalool oxide (1.5%), and (*E*)-nerolidyl acetate (1.5%)	Antibacterial (*Escherichia coli* and *Klesbsiella pneumoniae* MIC > 10 µL/mL; *Enterococcus faecalis* and S*taphylococcus epidermidis* MIC 5 µL/mL, *S. aureus* and *Streptococcus pyogenes* MIC 1.3 µL/mL, agar disk diffusion method)	[33]
*A. rosaeodora*	Adolpho Ducke Forest Reserve, Manaus, AM, Brazil	Stem	Linalool (86.0%), caryophyllene oxide (2.8%), and *trans*-linalool oxide (1.5%)	Antioxidant (EC_50_ 733 µg/mL, DPPH method); Antiplatelet activity (5.19%)	[70]
*A. rosaeodora*	Curuá-una, Santarém, PA, Brazil	Leaf	Linalool (88.6%), *cis*-linalool oxide (1.5%), and (*E*)-nerolidyl acetate (1.5%)	Isolated linalool at 0.1 µL/mL, light sedation (61.8 s), deep sedation (92.1 s) and deep anesthesia (464.3 s)	[71]
*A. rosaeodora*	Adolpho Ducke Forest Reserve, Manaus, AM, Brazil	Leaves and branches	Linalool (93.60%), α-terpinolene (3.37%), *cis*-linalool oxide (3.03%) and standard linalool	Antibacterial activity (MIC to EO 250–450 µg/mL and to standard linalool 550–650 µg/mL, disk-diffusion method); antioxidant activity (IC_50_: 15.46 μg/mL, ABTS method); trypanocide (*Trypanosoma cruzi*, IC_50_ to EO 150.5–911.6 µg/mL and to standard linalool 198.6–249.6 µg/mL); cytotoxicity (peritoneal macrophages from Balb/C mice, CC_50_ > 1000 µg/mL, MTT assay).	[81]
*A. rosaeodora*	Adolpho Ducke Forest Reserve, Manaus, AM, Brazil	Stem	Linalool (93.60%), α-terpinolene (3.37%), and *cis*-linalool oxide (3.03%)	Antibacterial activity (*E. coli* and *S. aureus*, MIC 200 and 150 µg/mL, respectively, disk-diffusion method); toxicological (*Artemia salina*, LC_50_: 282 mg/L).	[82]
*A. rosaeodora*	Zoobotanical Park of Emilio Goeldi Museum, Belém, PA, Brazil	Trunk wood	Linalool (84.8%), α-terpineol (2.9%), and geraniol (1.0%)	Relaxant and anticonvulsant (inhibition of cAMP, EO, IC_50_ 130 µg/mL; 3*R*-(−)-linalool, IC_50_ 310 µM; (±)-linalool, IC_50_: 300 µM)	[90]
			Mixture racemic, linalool: 3*R*-(–)-linalool (50.62%) and 3*S*-(+)-linalool (49.38%)		
*A. rosaeodora*	Zoobotanical Park of Emilio Goeldi Museum, Belém, PA, Brazil	Trunk wood	Linalool (84.8%), α-terpineol (2.9%), and geraniol (1.0%)	Antibacterial (*Escherichia coli,* EO at 0.3% chicken gastrointestinal tract)	[91]
*A. rosaeodora*	Jatapu River, Novo Airão, AM, Brazil	Trunk wood	Linalool (87.7%), α-terpineol (3.1%), *trans*-linalool oxide (1.5%), and *cis*-linalool oxide (1.5%)	Sedative potential (Male Swiss albino mice) EO at 100-300 mg/kg; EO at 2 and 100 µg/mL, percentages of blocking effect sciatic nerves from 75 to 95%	[92]
*A. rosaeodora*	Adolpho Ducke Forest Reserve, Manaus, AM, Brazil	Stem	Linalool (63.16%), *trans*-linalool furanoid (9.73%) and *cis*-linalool furanoid (7.69%)	Antioxidant activity (IC_50_: 48.67 and 40.06 µg/mL, ABTS and DPPH methods, respectively); toxicological (*Artemia salina*, LC_50_ 282-582 µg/mL; *Aedes aegypti*, LC_50_ 41.07 µg/mL)	[93]
*A. rosaeodora*	Maués, AM, Brazil	Leaf	Linalool (90.5%), synthetic linalool and isolated linalool	Anesthetic potential to the fish species *Colossoma macropomum*. EO at 0.050 µL/mL light sedation (68.3 s), deep sedation (204.1 s) and deep anesthesia (636.4 s)	[118]
*A. rosaeodora*	Maués, AM, Brazil	Leaf	Linalool (90.5%), synthetic linalool and isolated linalool	Synthetic linalool at 0.1 µL/mL, light sedation (32.7 s), deep sedation (68.9 s) and deep anesthesia (198.7 s)	[118]
				Isolated linalool at 0.1 µL/mL, light sedation (61.8 s), deep sedation (92.1 s) and deep anesthesia (464.3 s)	
*A. rosaeodora*	Zoobotanical Park of Emilio Goeldi Museum, Belém, PA, Brazil	Trunk wood	Not reported	Antiviral (Avian metapneumovirus, EC_50_: 20.86 µg/mL); cytotoxicity, cells derived from bovine kidney (CRIB), chicken embryo (CRER), mouse fibroblast cell (L929) and feline kidney cell lines (CRFK) CC_50_: 104.8 µg/mL	[120]
*A. rosaeodora*	Jatapu River, Novo Airão, AM, Brazil	Trunk wood	Linalool (87.7%), α-terpineol (3.1%), *trans*-linalool oxide (1.5%), and *cis*-linalool oxide (1.5%)	Cardiovascular (Male Wistar rat model), hypotension and bradycardia, EO at 10–20 mg/kg; vasorelaxant effects, IC_50_: 72.35 µg/mL	[121]

DPPH: 2,2-Diphenyl-1-picrylhydrazyl; LEC: lowest effective concentrations; MTT: 3-(4,5-dimethylthiazol-2-yl)-2,5-diphenyltetrazolium bromide; SPF: sunscreen protection factor.

### 7.2. Antifungal Activity

The fungistatic properties of oils from *Aniba* were tested by the disk diffusion method. The trunkwood oil of *A. canelilla* was evaluated against the human pathogenic fungi *Candida albicans*, *C. krusei*, and *C. parapsilosis*. oil (5 mg/mL) was active against these fungi with a halo of inhibition 25 mm to *C. krusei* and *C. albicans* and 18 mm to *C. parapsilosis*. The oil’s main compounds were 1-nitro-2-phenylethane (73.0%), methyleugenol (19.2%), and safrole (3.7%). MIC values and standard controls were not reported [77]. 

Different oil concentrations of *Aniba duckei* branches were tested on the growth of phytopathogenic fungi *Colletotrichum gloesporioides* and *Fusarium oxysporum*, displaying a mycelial inhibition of 65.0% and 72.09%, respectively, at 2%, and 100% inhibition for both fungi, at 0.4%. The oil was dominated by linalool (93.60%) with lesser amounts of *cis*-linalool oxide (3.03%) and α-terpinolene (3.37%). A linalool experiment itself supported its influence on the antifungal activity with a mycelial inhibition of 100% at a concentration of 0.2% [29]. 

Two Aniba oils from Adolpho Ducke Forest Reserve (AM, Brazil) were evaluated against the phytopathogenic fungi *Aspergillus flavus*, *A. niger*, *Fusarium oxysporum*, *F. solani*, *Alternaria alternata*, *Colletotrichum gloeosporioides*, *C. musae*, and *C. guaranicola*. The leaf oil from *A. canelilla* was mainly composed of 1-nitro-2-phenylethane (87.34%) and ethyl butanoate (3.1%), and the leaf oil from *A. parviflora* showed linalool (40.02%), caryophyllene oxide (4.33%), 1,8-cineole (4.02%), and β-phellandrene (4.01%) as major constituents. *Aniba canelilla* oil displayed strong activity against *C. guaranicola* (MIC, 0.15 μL/mL) and *A. niger* (MIC, 0.3 μL/mL), equivalent to Mancozeb (MIC, 0.3 μL/mL) used as a positive control. In addition, the activity was considered significant (MIC, 0.62 μL/mL) against *F. solani*, *F. oxysporum*, *C. gloeosporioides*, *C. musae*, and *A. flavus*, and low activity (MIC, 5 μL/mL) against *A. alternata*. *Aniba parviflora* oil exhibited higher activity (MIC: 0.62 μL/mL) against *A. flavus*, *F. solani*, and *F. oxysporum*, while to *A. niger*, *C. guaranicola*, *C. gloeosporioides*, *A. alternata*, and *C. musae*, the MIC values varied from 1.25 to 5.0 μL/mL. The effects of oils at 5 μL/mL on conidial germination of phytopathogenic fungi also were evaluated and displayed an inhibition rate varying from 83.5% to 96.7% in *A. canelilla* and 69.9% to 85.3% in *A. parviflora* [35].

The essential oils of leaves and branches of *A. rosaeodora* collected in Manaus (AM, Brazil) in different seasons were dominated by linalool in the leaves (69.0–71.0%) and in the branches (78–84%). The samples and racemic linalool (Sigma-Aldrich, St. Louis, MO, USA) exhibited antifungal activity against phytopathogenic fungi. All oils showed significant activity against *C. guaranicola*, *Colletotrichum* sp., *C. gloeosporioides* and *A. alternata* with MIC values ranging from 0.62 to 5.0 μL/mL [28].

### 7.3. Anti-Inflammatory and Antinociceptive Activities

The antinociceptive activity of 1-nitro-2-phenylethane, isolated from the bark and wood oils of *A. canellila*, was evaluated using three different rodent (Swiss mice) models of pain: the acetic acid-induced writhing test, the hot-plate latency test, and the formalin-induced inflammatory pain model. 1-Nitro-2-phenylethane significantly reduced the action of acetic acid used to induce writhing in mice at the dose of 15, 25, and 50 mg/kg. For the hot plate test, the administration of 1-nitro-2-phenylethane at 50, 100, and 200 mg/kg did not induce alterations in the latency time, compared to the morphine (10 mg/kg) positive control. The 1-nitro-2-phenylethane (25 and 50 mg/kg) was tested on two phases of pain model by formalin-induced pain, the early neurogenic and the second inflammatory through the administration of 20 μL of 1.0% formalin solution by intraplantar injection. The 1-nitro-2-phenylethane inhibited the licking response of mice in the second phase suggesting its antinociceptive effects [108]. The effect of 1-nitro-2-phenylethane was evaluated on paw and ear edema inhibition of male Swiss mice and Wistar rats. The anti-inflammatory potential was detected by reduced ear edema (73.8% and 79.4%), induced by croton oil (doses of 25 and 50 mg/kg, body wt.), in comparison to dexamethasone (positive control, 10 mg/kg), which reduced edema by 87.01%. The 1-nitro-2-phenylethane showed a dose-dependent effect on paw edema induced by dextran. At a dose of 25 mg/kg, 1-nitro-2-phenylethane reduced the edema by 15.58%, 26.78%, 44.92% and 30.07%, after 30, 60, 90, and 120 min. At a dose of 50 mg/kg, it showed inhibition of edema development of 38.1%, 61%, 69.09%, and 73.65%, for 30, 60, 90, and 120 min, respectively. Similarly, for paw edema induced by carrageenan, at a dose of 25 mg/kg, 1-nitro-2-phenylethane showed edema reduction of 26.83%, 43.91%, 41.6%, and 39.85% after 2, 3, 4, and 5 h. At a dose of 50 mg/kg, it inhibited 51.76%, 54.46%, 47.2%, and 49%, after 2, 3, 4, and 5 h, respectively [111].

### 7.4. Antioxidant and Photoprotective Activities

Essential oils have been recognized as natural antioxidants, because they contain compounds such as terpenoids and phenylpropanoids capable of reacting with radicals and reducing or neutralizing oxidative stress [122]. All *Aniba* oils evaluated below were tested by the 2,2-diphenyl-1-picrylhydrazyl (DPPH) or 3-ethylbenzothiazoline-6-sulfonic acid (ABTS) assays.

Two trunkwood oils of *A. canelilla* presented 1-nitro-2-phenylethane (92.1%, 70.2%) and methyleugenol (4.30%, 25.8%) as major compounds. These oils and pure isolated 1-nitro-2-phenylethane indicated weak activity, with EC_50_ values of 223.81 μg/mL, 172.52 μg/mL, 792.50 μg/mL, respectively, in comparison to Trolox (EC_50_ 4.67 μg/mL), the reference standard [27].

The antioxidant activities of *A. panurensis* and *A. roseaodora* leaf oils showed EC_50_ > 1000 μg/mL and 733 μg/mL, respectively, in comparison to quercetin at 3.13 μg/mL. *Aniba panurensis* oil exhibited β-caryophyllene (33.5%), germacrene-D (25.4%), and α-copaene (7.5%), and *A. rosaeodora* oil displayed linalool (86.0%), caryophyllene oxide (2.8%), and *trans*-linalool oxide (1.5%), as their main constituents [70].

The antioxidant activity of *A. rosaeodora* stem oil, collected at Adolpho Ducke Forest Reserve (AM, Brazil), containing linalool (63.16%), *trans*-linalool furanoid (9.73%), and *cis*-linalool furanoid (7.69%), was tested with the ABTS and DPPH assays. The oil showed IC_50_ values of 48.67 (ABTS) and 40.06 µg/mL (DPPH), respectively [93]. The *A. rosaeodora* leaves and thin branches oil, collected in Adolpho Ducke Forest Reserve (AM, Brazil), containing linalool (93.60%), α-terpinolene (3.37%), and *cis*-linalool oxide (3.03%), and the linalool standard were tested by the ABTS method. The effective concentration necessary to scavenge 50% of the ABTS (EC_50_) was 15.46 μg/mL for *A. rosaeodora* oil and 6.78 μg/mL for linalool isolated [81].

*Aniba parviflora* leaf and branch oils showed significant antioxidant activity at a concentration of 2.50 mg/mL, with TEAC values of 90.1–287.9 mgTE/mL for the leaves and 94.1–358.4 mgTE/mL for the branches. β-Phellandrene (15.1%), linalool (14.1%), and γ-eudesmol (12.9%) were the main compounds in the leaves, and γ-eudesmol (16.8%), β-caryophyllene (15.4%), linalool (12.4%), β-phellandrene (6.7%), and bicyclogermacrene (6.0%) in the branches [32].

The photoprotective capacity of *A. canelilla* leaf and branch oils was evaluated by spectrophotometric method, applying a wavelengths scan from 280 to 400 nm [123]. At a concentration of 1% in isopropanol, the oils collected during the dry and rainy seasons displayed solar protection factor (FPS) varying from 7.54 to 14.08 in the leaf oil and 5.49–6.93 in the branch oil. Quercetin (FPS 261.23), benzophenone (FPS 289.80), and commercial sunscreen (FPS 72.08) were used as standards [112]. According to Brazilian legislation, a product to be used in photoprotection cosmetics must have an SPF value of at least 6 [124].

### 7.5. Cardiovascular Activity

Cardiovascular effects of intravenous (i.v.) treatment with the essential oil of *A*. *canelilla* were evaluated in rodents. The main compounds of the oil were 1-nitro-2-phenylethane (52.4%), methyleugenol (38.6%), and selin-11-en-4α-ol (2.5%). Hypotensive effects of essential oil from *A. canelilla* bark were assessed in pentobarbital-anesthetized and conscious rats. Intravenous injections of EO (1 to 20 mg/kg) induced immediate and dose-dependent decreases in mean aortic pressure and heartbeat at doses of 1 and 5 mg/kg, respectively, in both experiments. The EO (100 μg/mL) also showed smooth-muscle relaxant activity in aorta preparations containing endothelium previously contracted with potassium (60 mM) [85].

Increasing concentration injections (1–10 mg/kg) of purified 1-nitro-2-phenylethane from the *A. canelilla* bark collected at Paragominas (PA, Brazil) caused dose-dependent hypotensive and bradycardic effects at a minimal concentration of 3 mg/kg in normotensive rats anesthetized with sodium pentobarbital (50 mg/kg). In aorta preparations containing endothelium, 1-nitro-2-phenylethane (1 to 300 μg/mL) exhibited vasorelaxant effects after phenylephrine-induced contraction in a concentration-dependent manner with IC_50_ value of 60.1 μg/mL [113].

Injections of essential oil (1–20 mg/kg) and 1-nitro-2-phenylethane (1–10 mg/kg), obtained from *A. canelilla* bark from Paragominas (PA, Brazil), elicited dose-dependent hypotensive and bradycardic effects in rats anesthetized with sodium pentobarbital (50 mg/kg). At 600 μg/mL of essential oil and 1-nitro-2-phenylethane, maximal relaxations in the superior mesenteric artery were previously contracted with phenylephrine [23].

The constituent 1-nitro-2-phenylethane (NPE), isolated in high-grade purity (98%) from the *A. canelilla* wood bark oil, collected at Paragominas (PA, Brazil), was investigated for its vasodilator effect in rat aorta, using isolated vessel bioassays. The NPE (0.7–1984.6 mM) relaxed the contractions of intact endothelium, induced by Hyp9 (100 mM) and phorbol 12,13-dibutyrate (1 mM), with IC_50_ values of 119.0 µM and 203.1 μM, respectively. In endothelium-intact mesenteric arterial rings, NPE (0.2–1984.6 μM) also relaxed sustained contractions, induced by norepinephrine (10 mM), with an IC_50_ value of 43.0 μM. Thus, it is suggested that NPE appeared to exert vasodilatory effects compatible with a drug’s profiles that induce stimulation and improve production in aortic tissues [114].

A study investigated the action of 1-nitro-2-phenylethane (NPE) (synthetic), the main constituent of *A. canelilla* oil, in the cardiovascular responses of spontaneously hypertensive rats (SHRs). Intravenous injections of oil (1–20 mg/kg) and NPE (1–10 mg/kg) elicited dose-dependent hypotensive and bradycardic effects. The vasorelaxant effect, induced by oil and NPE, was also tested in superior mesenteric artery from SHRs, at concentration 0.1–1000 μg/mL. Both oil and NPE relaxed superior mesenteric artery (SMA) preparations, pre-contracted with 75 mM KCl, with IC_50_ values of 294.19 and 501.27 μg/mL, respectively. The inhibitory effects of oil and NPE on contractions were induced by the exogenous addition of Ca^2+^ (75 mM) [24]. The mechanisms underlying the vascular effects of NPE were investigated in rat isolated thoracic aortic preparations, at concentration 0.1–100 μg/mL, in NPE relaxed endothelium-intact or endothelium-denuded aortic preparations pre-contracted with to KCl (60 mM) or phenylephrine (1 μM) [115].

The oil of leaves from *Aniba panurensis* (Meisn.) Mez and the oil of stems from *A. rosaeodora*, collected in Adolpho Ducke Forest Reserve (AM, Brazil), were assessed for their antiplatelet potential, using a method based on the measurement of platelet aggregates after exposure to the aggregating agent adenosine diphosphate (4.27 μg/mL). The oils at 1% were added to the platelet-rich plasma. *A. panurensis*, rich in β-caryophyllene (33.5%), germacrene-D (25.4%), and α-copaene (7.5%) showed less inhibition of 3.57%, while *A. rosaeodora*, dominated by linalool (86.0%), caryophyllene oxide (2.8%) and *trans*-linalool oxide (1.5%), exhibited inhibition of 5.19% in comparison to the standard acetylsalicylic acid (0.01%), with 100% inhibition [70].

Oil of trunkwood from *A. rosaeodora*, collected in Novo Airão (AM, Brazil), containing linalool (87.7%), α-terpineol (3.1%) and geraniol (1.2%), was evaluated regarding its cardiovascular effects in normotensive rats (male Wistar), anesthetized with sodium pentobarbital (50 mg/kg), treated with oil (1–20 mg/kg). Monitoring of cardiac effects was carried out by measuring blood pressure. The oil at 10 and 20 mg/kg induced two phases of hypotension and bradycardia. Initially, rapid bradycardia (1–2 s) occurred coincidentally (2–3 s) with arterial hypotension (phase 1), and then a delayed and more lasting decrease in blood pressure associated with second bradycardia (phase 2). In aortic preparations with intact endothelium, the oil (0.15–771.25 μg/mL) relaxed the phenylephrine-induced contractions (IC_50_ 95.08 μg/mL). The oil-induced vasorelaxant effects were reversible after wash and remained unaffected by the endothelium removal (IC_50_ 72.35 μg/mL) [121].

### 7.6. Cytotoxic Activity

The cytotoxic properties of some *Aniba* oils were also evaluated using the MTT method. The *A. canelilla* leaf oil from Adolpho Ducke Forest Reserve (AM, Brazil) showed 1-nitro-2-phenylethane (88.9%) and β-caryophyllene (4.21%) as the major components. The specimen showed low cytotoxicity against murine peritoneal macrophages from BALB/c mice, with IC_50_ 9.3 μg/mL, compared to the standard pentamine (IC_50_ 24.4 μg/mL) [22].

The *A. canelilla* stem oil, containing 1-nitro-2-phenylethane (83.68%) and methyleugenol (14.83%), was evaluated against human lymphocytes for 24 h and 48 h. The oil at different concentrations (0.5%, 1.0% and 2.0%) did not show significant cytotoxic effects. However, after 24 h at 2.0%, the mixture of the isolated main constituents (1:1) showed cell viability of 78.65% and the isolated 1-nitro-2-phenylethane and methyleugenol of 95.51% and 89.2%, respectively [86].

*Aniba parviflora* oils, rich in β-phellandrene (15.1%), linalool (14.1%), and γ-eudesmol (12.9%) in the leaves, and γ-eudesmol (16.8%), β-caryophyllene (15.4%), and linalool (12.4%) in the branches, were evaluated against human breast adenocarcinoma cells line MCF-7. The leaf and branch oils showed good antiproliferative activity with IC_50_ values of 67.9 and 102.2 μg/mL, respectively. Dimethyl sulfoxide was used as the negative control and tingenone (100 μg/mL) as the positive control [32].

The cytotoxicity of *A. rosaeodora* wood oil was evaluated through the maximum non-toxic concentration (MNTC). The analysis was determined microscopically by observing cell morphological changes at 24, 48, and 72 h of incubation, followed by MTT assay. The cells used were bovine kidney CRIB, chicken-embryo related CRER, mouse fibroblast cell L929, and feline kidney cell lines CRFK. The oil showed 50% cytotoxic concentrations (CC_50_) of 104.8%, with a selectivity index of 5. The oil composition was not reported [120]. The *A. rosaeodora* leaf and thin branch oil containing linalool (93.60%), α-terpinolene (3.37%), and *cis*-linalool oxide (3.03%), linalool standard (1000–7.8 μg/mL) and the positive control benznidazole (200–0.78 μg/mL) were evaluated by MTT method on cell viability of peritoneal macrophages from Balb/C mice. The oil and linalool did not exhibit cell toxicity at the highest concentration analyzed (CC_50_ > 1000 μg/mL) [81].

The *A. parviflora* bark oil, collected in Curuá, municipality of Santarém (PA, Brazil), containing linalool (16.3%), α-humulene (14.5%), δ-cadinene (10.2%), α-copaene (9.51%), and germacrene B (7.58%), was evaluated on the growth of human hepatocellular carcinoma cells in the culture and in the development of tumors in a xenograft model. The oil was selective for HepG2 cells with IC_50_ values of 9.05 μg/mL. Based on their bibliographic survey, the authors considered essential oils with IC_50_ values < 30 μg/mL the most promising for the development of cytotoxic drugs in cancer therapy. With respect to the development of tumors, the animals treated with the oil showed a reduction in tumor weights 0.40 g and 0.17 g at the 40 and 80 mg/kg doses of oil [119].

### 7.7. *Nervous System* *Activity*

Essential oils and their components can induce innumerable physiological actions in the central nervous system, such as analgesic, anxiolytic, relaxing, sedative, and behavior and perception effects, in addition to the treatment of epilepsy and degenerative diseases such as Alzheimer’s and Parkinson’s diseases [125].

The *A. canelilla* trunkwood oil, presenting 1-nitro-2-phenylethane (70.2%) and methyleugenol (25.8%), has displayed acetylcholinesterase inhibitory properties by the bioautography method using the Fast blue salt B as the reagent. The oil and 1-nitro-2-phenylethane (98.0%) showed a value to the detection limit (DL) equivalent to physostigmine (0.01 ng), an alkaloid used as the positive control [116].

The *A. rosaeodora* and *A. parviflora* leaf oils and linalool standard 97% were evaluated in the central nervous system of rodents, employing neurobehavioral tests. The spontaneous locomotion was smaller in the group treated with 3.5 mg/kg of *A. rosaeodora* oil when compared with the non-treated control group. In the depressive type method, the *A. rosaeodora* oil (35 mg/kg) and linalool (30 mg/kg) caused a reduction in the latency period and an increase in the self-cleaning time, a similar behavior was noted for the control group, fluoxetine (10 mg/kg). Both oils and linalool standard significantly decreased the immobility time of the animals when compared to the positive control fluoxetine. The major components of the oil from *A. rosaeodora* were linalool (88.6%), while in *A. parviflora* were linalool (45.0%), β-phellandrene (17.3%), and α-phellandrene (4.1%) [71].

The oils from leaves of *A. rosaeodora* (linalool, 90.5%) and *A. parviflora* (linalool 29.6%, β-caryophyllene, 10.9%, and α-phellandrene, 10.5%), standard linalool 97%, and linalool isolated from the oil of *A. rosaeodora* were evaluated as anesthetics in young *Colossoma macropomum* fish. At concentrations of 0.025 and 0.05 μL/mL, the *A. rosaeodora* oil was twice as efficient in light sedation (123.0 s, 68.3 s), deep sedation (355 s, 204 s), and deep anesthesia (636.4 s) compared to *A. parviflora* oil, and standard and isolated linalool, which needed two-fold concentrations to provoke the same effects. Fish exposed to 0.05–0.2 μL/mL of *A. rosaeodora* oil, 0.1–0.3 μL/mL of *A. parviflora* oil, and both linalool samples reached deep anesthesia 1–10 min. The induction time for all anesthesia stages decreased with the increasing concentration of the anesthetics. The isolated linalool showed the lengthier time for anesthesia induction in some stages and recovery at 0.1 and 0.2 μL/mL, in comparison to standard linalool [118]. (3*S*)-(+)-Linalool and (3*R*)*-*(–)-linalool have different properties on the central nervous system, related to depressant effects, analgesic and anti-inflammatory activities [126].

The sedative effects of *A. rosaeodora* trunk wood oil in rats and mice were investigated and showed decreased latency and increased duration of sleeping time at doses of 200 and 300 mg/kg. On the other hand, the combination of the oil (100 mg/kg) and the sedative agent pentobarbital (40 mg/kg) increased the action. The blocking effect of oil for 30 min on rat sciatic nerves from 75.0% at 2 μg/mL to 95.0% at 100 μg/mL was irreversible. The main compounds of the EO were linalool (87.7%), α-terpineol (3.1%), *trans*-linalool oxide (1.5%), and *cis*-linalool oxide (1.5%) [92].

Relaxant and anticonvulsant activities on the central nervous system of *A. rosaeodora* wood oil and linalool were evaluated on adenylate cyclase activity (an enzyme that catalyzes cAMP hydrolysis) in a chick retina model. The decreased levels of cAMP protect against seizures in a variety of epilepsy models. The cAMP accumulation was stimulated by forskolin (10 μM), and inhibited by the EO (6 and 17.5 mM). The effects were also evaluated in the presence of the 3-isobutyl-1-methylxanthine (500 μM), an inhibitor of cAMP, which did not interfere with the positive effects of the EO (1–6 mM) on cAMP production. The oil, (3*R*)-(–)-linalool and racemic (±)-linalool displayed IC_50_ values of 130, 310, and 300 μM, respectively. The inhibition of cAMP takes part in the molecular mechanisms underlying the relaxant and anticonvulsant effects of EO and linalool in the central nervous system. The trunk wood of *A. rosaeodora* collected in Belém (PA, Brazil) was mainly composed of linalool (84.8%), α-terpineol (2.9%), and geraniol (1.0%). Its enantiomeric distribution of linalool was analyzed by GC chiral column and revealed a nearly racemic mixture with the proportion 50.62% of (3*R*)-(–)-linalool and 49.38% of (3*S*)-(+)-linalool [90].

### 7.8. Toxicological Studies

The toxicity of oils from *A. canelilla* trunkwood, presenting 1-nitro-2-phenylethane (92.1%, 70.2%) and methyleugenol (4.30%, 25.8%), as well as isolated 1-nitro-2-phenylethane, were evaluated by the brine shrimp (*Artemia salina*) lethality test and showed a median lethal concentration value for the oils (LC_50_, 21.61 μg/mL) and 1-nitro-2-phenylethane (LC_50_, 20.37 μg/mL). DMSO was used as negative control [27]. Essential oils with LC_50_ values equal to or higher than 250 μg/mL are considered non-toxic against *A. salina* [127], and LC_50_ below 50 μg/mL have highly efficient larvicidal effects [128]. The *A. canelilla* leaf oil collected in Adolpho Ducke Forest Reserve (AM, Brazil), containing 1-nitro-2-phenylethane (88.9%), β-caryophyllene (4.21%), and β-phellandrene (0.80%) was evaluated against *A. salina*. The oil indicated low toxicity (LC_50_ 68.37 μg/mL). DMSO was used as negative control (LC_50_ > 1000 μg/mL) and lapachol as positive control (LC_50_ 23.0 μg/mL) [22].

*Aniba rosaeodora* stem oil, collected in Adolpho Ducke Forest Reserve (AM, Brazil), containing linalool (63.16%), *trans*-linalool furanoid (9.73%), and *cis*-linalool furanoid (7.69%), was tested for toxicity using the brine shrimp lethality assay and presented LC_50_ values varying from 282 to 582 μg/mL. Potassium dichromate was used as the positive control [93]. *Aniba duckei* branch oil, at a minimal concentration (2 μg/mL), showed 100% larvicidal activity against *Artemia franciscana*, and at a maximal concentration (10 μg/mL), all larvae died after just 10 min. The oil composition was not reported [35].

Essential oils of *Aniba* species were also tested against the larvae of *A. aegypti*. *Aniba rosaeodora* stems oil showed LC_50_ 41.07 μg/mL, and phosphorothioate at 100 ppm was used as the positive control [93]. *Aniba duckei* stem oil exhibited LC_90_ 54,000 μg/mL and LC_50_ 2200 μg/mL against the larvae of *A. aegypti* [117]. A review carried out with 361 essential oils from 269 plants concluded that essential oils are active against *A. aegypti* larvae with LC_50_ < 100 mg/L [128]. The toxicological potential of *A. duckei* branch oil and the standards (3*R*)-(–)-linalool and (±)-linalool were evaluated against *Aedes aegypti* larvae. The branch oil was composed of linalool (89.34%), α-terpineol (3.06%), and *cis*-linalool oxide (1.94%). The best larvicidal activity was detected for the oil (LC_50_ 250.61 μg/mL), while (3*R*)-(–)-linalool and racemic (±)-linalool showed LC_50_ values of 279.89 μg/mL and 346 μg/mL, respectively. The organophosphate Temephos (100 ppm) and mineral water containing 0.04% Tween (20 mL) were used as the positive and negative control, respectively [81].

The toxicity of *A. rosaeodora* stem oil, presenting linalool (93.60%), α-terpinolene (3.37%), and *cis*-linalool oxide (3.03%), was evaluated by the *Artemia salina* lethality test and showed an LC_50_ 282 mg/L [82], classified as nontoxic [127].

### 7.9. Other Activities

The *A. canelilla* leaf oil, dominated by 1-nitro-2-phenylethane (88.9%), inhibited promastigotes of *Leishmania amazonensis*, the etiological agent of leishmaniasis, with an IC_50_ value of 40 μg/mL, in comparison to pentamidine (IC_50_ 4.80 μg/mL), the reference drug [22].

The anti-trypanosomal activity of *A. canelilla* stem EO, rich in 1-nitro-2-phenylethane (83.68%) and methyleugenol (14.83%), was evaluated against *Trypanosoma evansi*. The assays were performed using the oil, the two main isolated constituents, and a mixture (1:1) at concentrations ranging from 0.5 to 2.0%. The tested oil presented a trypanocidal profile similar to the positive control, diminazene aceturate (0.5%). After 6 h, no parasites were found alive (complete motility cessation) in all oil concentrations tested. The compound 1-nitro-2-phenylethane (0.5%) was able to reduce the number of live trypanosomes to zero after only 3 h; methyleugenol and the mixture (2.0%) caused the death of the trypanosomes after 1 h [86].

The antiviral activity of oil from *A. rosaeodora* trunkwood, collected at Zoobotanical Park of the Emilio Goeldi Museum, located in Belém (PA, Brazil), showed cytopathic effects through visual microscopic analysis and inhibited the viral growth of avian metapneumovirus (EC_50_: 20.86 μg/mL). The oil composition was not reported [120].

The leaves and thin branches oil from *A. rosaeodora*, collected at Adolpho Ducke Forest Reserve (AM, Brazil), containing linalool (93.60%), α-terpinolene (3.37%), and *cis*-linalool oxide (3.03%), were evaluated against epimastigote and intracellular amastigote forms of *Trypanosoma cruzi*, as well as the linalool standard. The oil showed the IC_50_ values 150.5 μg/mL and 911.6 μg/mL for epimastigotes and intracellular amastigotes, respectively, while the linalool IC_50_ values ranged from 198.6 to 249.6 μg/mL and benznidazole, the positive control, from IC_50_ 1.805 to 0.482 μg/mL for both forms, respectively [81].

### 7.10. Biological Activities from Commercial Aniba Rosaeodora Essential Oils

Although most biological activities reported for commercial *A. rosaeodora* oils do not describe the plant’s part or chemical composition, it is essential to know the wide application given to them. The antimicrobial activity of oils obtained from Sunspirit Oils Pty Ltd., Australia, was evaluated by agar dilution and broth microdilution methods and exhibited activity against *Acinetobacter baumanii*, *Aeromonas sobria*, and *E. coli* (MIC 1.2%), *Salmonella typhimurium*, *S. aureus*, and *C. albicans* (MIC 0.25%), *E. faecalis*, *K. pneumoniae* and *Serratia marcescens* (MIC 0.5%) using agar dilution assay; Mueller Hinton agar, with 0.5% (*v/v*) tween-20 was used as positive growth control. However, the assays performed by the microdilution method against *C. albicans*, *E. coli* and *S. aureus* showed MIC values of 0.12%, the EO composition and standard were not mentioned. The results obtained by each of these methods may differ as many factors vary between assays [129,130], these include differences in microbial growth, exposure of micro-organisms to the oil, the solubility of oil or oil components, and the use and quantity of an emulsifier. These and other elements may account for the significant differences in MICs obtained by the agar and broth dilution methods in this study [131].

A rosewood oil sample purchased from Erbamea (Istrana, Treviso, Italy), containing linalool (60.1%), limonene (19.2%), geraniol (7.8%), and cymene (4.1%) showed antibacterial activity by the broth microdilution method. The MIC values were 250 μg/mL to *Bacillus cereus* and *A. baumannii*, 500 μg/mL to *B. subtilis*, *S. aureus* and *E. coli*, and 2000 µg/mL to *Serratia marcescens* and *Yersinia enterocolitica*. In addition, the combination of the oil with the drug gentamicin was evaluated for its synergistic effect. The interaction was defined quantitatively as a fractional inhibitory concentration (FIC). the synergism is indicated when FIC values are below 0.5. The oil in association with gentamicin revealed a strong synergistic mode of action. MIC values were reduced to an interval varying from 10 to 100 μg/mL. Mueller Hinton Broth was used as positive growth control [95]. An *A. rosaeodora* wood oil, commercially obtained from Brazil, was mainly composed of linalool (80.0%) and exhibited antibacterial activity against *B. cereus, Micrococcus luteus, Alcaligenes faecalis*, and *P. aeruginosa*, with inhibition zones varying from 12 mm to 19 mm, and against *S. aureus, S. faecalis,* and *Enterobacter cloacae* with inhibition zones from 5 mm to 7 mm. The same oil indicated antifungal potential against *C. albicans* and *Aspergillus niger*, with inhibition zones of 33 mm and 32 mm, respectively, and *Rhizopus oligosporus* with only a 2 mm inhibition zone. The assays were performed by the disk diffusion method. The origin of the sample, MIC values, and reference standards was not reported [132]. Rosewood oil sample purchased from Stony Mountain Botanicals, Ltd. (Loudonville, OH, USA) was evaluated against *Aeromonas salmonicida* subsp. *salmonicida*, a bacterium that causes fish furunculosis, by the disk diffusion method. The inhibition zone of the oil was 16.7 mm, which is considered a moderate inhibition. The MIC value was not determined [133]. The diameter of inhibition zones, including the disc diameter, is considered as weak (10–13.9 mm), moderate (14–18 mm), or strong (>18 mm), according to [107]. Another rosewood oil sample, purchased from Anthémis Aromatherapie (Oosterstreek, The Netherlands), was evaluated by the broth dilution method against *B. cereus* and showed MIC value 1.0% to vegetative cells and MIC value >1.00% to spore germination [134].

The antifungal activity of a rosewood oil sample obtained from the Institute for Medicinal Plant Research “Dr. Josif Pancic”, Serbia, containing 81.27% of linalool, was evaluated by the disk diffusion method. The inhibition of mycelial growth and inhibition of spore germination was performed by macro-dilution and micro-dilution assays. The oil was active against all fungi in the micro-dilution method, with MIC value from 1 to 10 μL/mL for *Alternaria alternata*, *Aureobasidium pullulans*, *Cladosporium cladosporioides*, *C. fulvium*, *Fusarium tricinctum*, *F. sporotrichoides*, *Phomopsis helianthin*, and *Phoma macdonaldii*. Meanwhile, in the macro-dilution method, the MIC ranged from 0.5 to 7.5 μL/mL for *A. alternata*, *A. pullulans*, *C. fulvium*, *P. helianthin*, and *P. macdonaldii*. Bifonazole was used as a positive control [94]. Rosewood oil samples obtained commercially in Pretoria and Johannesburg, South Africa, were tested against *Geotrichum citri-aurantii*, a postharvest pathogen of *Citrus*, by incorporating 0.5 μL/mL of oil into the culture medium, and showed mycelial growth inhibition of 12.1%, which was considered low. Kenopel ^®^200SL (1 μL/mL) was used as positive control [135].

The nematicidal activity from *A. rosaeodora* oil purchased from Berje (Bloomfield, NJ, USA) was evaluated against *Bursaphelenchus xylophilus* by immersion bioassay during a 24-h exposure. The oil at 10 mg/mL had a significant lethal activity of 94% mortality and toxicity LC_50_ 2.99 mg/mL. Ethanol-Triton X-100 solution was used as control, and fenitrothion was used as a standard nematicide but was ineffective (LC_50_ > 10 mg/mL) [136]. The lethal activity was considered strong, with mortality above 80% [137].

The cytotoxic potential of commercial *A. rosaeodora* oil was also evaluated. A sample of unknown origin containing linalool (80%) and α-terpineol (4.5%) was tested against human epidermoid carcinoma cell line (A431), human epidermal keratinocytes CRL-2404 (HEK001), immortalized HaCaT cells (HaCaT), and on normal primary human epidermal keratinocytes (NHEK). The MTT assay showed a reduction in cell viability observed in A431 and HaCaT cells (<20% viability) at 0.4 μL/mL of EO for 4 h, whereas HEK001 and NHEK cells were much less affected (>70% viability), the prolonged incubation for 12 h in the HEK001 and NHEK cells reduced viability to approximately 50%. The EO triggered the production of reactive oxygen species, induced depolarization of the mitochondrial membrane, and caused caspase-dependent cell death [97].

The insecticidal and larvicidal effects of commercial samples of rosewood oil have also been evaluated. A sample obtained from the Fragrance and Flavour Development Center, India, was tested as a repellent against *A. aegypti* using the cage and cone bioassay methods. The oil was effective as a repellent until 1.5 h, compared with synthetic repellents *N*,*N*-diethyl-*m*-toluamide (DEET), and *N*,*N*-diethylphenylacetamide (DEPA), used as positive controls, and provided complete protection ranging from 5 to 6 h. The EO exhibited 10%, 66%, and 100% knockdown effects at 0.1%, 1%, and 5%, respectively, and showed a practical knockdown dose value (KT_50_) of 2.029%. Malathion and acetone were used as positive and negative controls, respectively. The gas chromatograph coupled-electroantennogram detection showed that linalool and oxide linalool elicited a spick response in the antenna of *A. aegypti* female mosquito. However, the concentrations of these compounds were not mentioned [138]. A sample obtained from Edens Garden, San Clemente (CA, USA) was evaluated as a vapor for enhancement of deltamethrin efficacy in pyrethroid-susceptible and resistant strains of the *A. aegypti* mosquito. Vapor bioassays were made by exposing mosquitoes to the vapor of essential oil (100 μL), which showed 48.33% mortality, ([139]). The larvicidal activity of EO from the heartwood of *A. rosaeodora* obtained from Guangzhou Yuxitang Cosmetics Co., Ltd. (China) was tested against *Aedes albopictus* larvae at 100 ppm the EO showed 5.0% of mortality [140].

The oil purchased from Oshadhi Ltd. (Cambridge, England) containing linalool (44.4%), linalyl acetate (31.4%), and β-caryophyllene (10.5%), and pure linalool (97%, Sigma-Aldrich, St. Louis, MO, USA), were evaluated against the adzuki bean beetle (*Callosobruchus chinensis*). For this study, 10 and 100 ng of oil and the pure linalool per filter paper strip were submitted for the repellence test and 0–20 μL of oil to oviposition assays. Only the pure linalool exhibited male insect repellency in higher concentrations (100 ng), and neither pure linalool nor the oil was effective oviposition deterrent [96].

*Aniba rosaeodora* oil, purchased from a commercial company with unknown origin, was tested regarding its anesthetic efficacy in goldfish (*Carassius auratus*). Linalool was the main compound from the oil (86.23%), followed by *cis*-linalool oxide (1.06%) and β-selinene (0.95%). The lowest effective concentration (LECs) for the oil was 0.25 μL/mL, which showed rapid induction and recovery of anesthetic effect. No mortality or adverse effects occurred with the fish. Thus rosewood oil was considered a new potential anesthetic agent for fish species [98].

A study showed the comparative effects of (3*S*)-(+)-linalool, (3*R*)-(–)-linalool, and (±)-linalool on the behavioral parameters of anticonvulsant drugs in mice. The (±)-linalool (200 mg/kg) was more effective than (3*S*)-(+)-linalool and (3*R*)-(–)-linalool because it increased the latency of convulsions in a pentylenetetrazole-induced seizure model, showing the synergistic action of its constituents. In the picrotoxin-induced seizure model, both (3*R*)-(–)-linalool and (±)-linalool presented activity at the dose of 200 mg/kg. When evaluated at maximal electroshock-induced seizure models, (3*S*)-(+)-linalool, (3*R*)-(–)-linalool and (±)-linalool decreased the convulsion time of the mice in the doses of 200 and 300 mg/kg [141].

## 8. Conclusions

The genus *Aniba* has a predominantly Amazonian distribution and many species have been used as traditional herbal medicines. This review demonstrated the high chemical and biological potential of essential oils from their species. The multivariate analysis of the chemical classes present in the essential oils allowed the identification of chemical markers, which contributed to fill the morphological and phylogenetic gaps of the genus. The benzenoids and phenylpropanoid were well represented for *A. canelilla*, *A. guianensis*, *A. gardineri* and *A. puchury-minor* species. On the other hand, *A. rosaeodora* and *A. duckei* were characterized by the high concentration of oxygenated monoterpenes. The oils of *A. fragrans*, *A. gardneri*, *A. hostmanniana*, *A. parviflora*, *A. riparia*, *A. parviflora*, *A. rosaeodora*, *A. terminalis*, and *A. puchury-minor* showed significant chemical diversity for their main compound classes such as terpenoids, benzenoids, and phenylpropanoids.

*Aniba* essential oils and their compounds have a wide range of pharmacological activities: 1-Nitro-2-phenylethane, a major component in *A. canelilla essential* oils, is responsible for the cinnamon-like odor of the plant, and has shown hypnotic, anticonvulsant, anxiolytic, vasorelaxant, hypotensive, and anti-inflammatory activities. The essential oils of *A. rosaeodora*, *A. duckei*, *A. fragrans* and *A. parviflora* are rich in linalool, which give these species a floral-like odor. Linalool has shown antimicrobial, antiparasitic, anti-inflammatory, and central nervous system effects. Both enantiomers of linalool have shown anxiolytic and anticonvulsant effects, but (3*R*)-(–)-linalool is apparently more active than (3*S*)-(+)-linalool in terms of sedative activity. The high concentrations of linalool or 1-nitro-2-phenylethane in *Aniba* essential oils likely account for the traditional uses of these plant species as well as the biological activities of the oils.

## Data Availability

The data presented in this study are available on request from the corresponding author.
